# Characterization of SPTLC2 as a key driver promoting microglial activation and energy metabolism reprogramming after ischemic stroke through bulk and single-cell analyses combined with experimental validation

**DOI:** 10.1007/s10565-025-10085-9

**Published:** 2025-10-07

**Authors:** Yongxing Lai, Peiqiang Lin, Zhiyun Wu, Tin Chen, Wenyao Hong, Mouwei Zheng, Jianhao Chen, Nan Liu, Hongbin Chen

**Affiliations:** 1 Department of Neurology, Fujian Medical University Union Hospital, 29 Xinquan Road, Fuzhou, 350001 Fujian China; 2 Department of Geriatric Medicine, Fuzhou University Affiliated Provincial Hospital, Fuzhou, China; 3 Department of Neurology, Fuzhou University Affiliated Provincial Hospital, Fuzhou, China; 4 Department of Rehabilitation Medicine, Fuzhou University Affiliated Provincial Hospital, Fuzhou, China; 5 Department of Neurosurgery, Fuzhou University Affiliated Provincial Hospital, Fuzhou, China; 6 Institude of Clinical Neurology, Fujian Medical University; Clinical Research Center for Precision Diagnosis and Treatment of Neurological Diseases of Fujian Province , Fuzhou, 350001 China

**Keywords:** Stroke, Single-cell RNA sequencing, Mitochondria, Machine learning, SPTLC2, Microglia

## Abstract

**Background:**

Ischemic stroke (IS) stands as a principal contributor to high rates of sickness and death. The condition's pathological development is complicated, featuring mechanisms like mitochondrial impairment and the activation of microglial cells. A thorough grasp of these intricate processes is vital for creating successful treatment strategies.

**Methods:**

We applied Weighted Gene Co-expression Network Analysis (WGCNA) to find gene sets with a strong correlation to IS. Integrated machine learning approachs were used to identify key mitochondrial-related genes (MRGs). From this analysis, SPTLC2 was identified as a pivotal MRG and was subsequently analyzed in detail using single-cell RNA sequencing (scRNA-seq) datasets. We performed functional confirmation using experimental stroke simulations, which included transient middle cerebral artery occlusion (tMCAO) in mice and in vitro oxygen–glucose deprivation/reoxygenation (OGD/R) on primary microglia.

**Results:**

WGCNA revealed two critical modules (yellow and blue) comprising 5348 genes, which were predominantly enriched in immune response, nerve regeneration, and lipid metabolism. We exhibited the robust and superior performance of MRGs in stroke prediction, which contributed to an optimal combination of ridge regression and random forest fitted on 18 MRGs. Subsequently, elevated expression of the SPTLC2 gene was observed in microglia following stroke. Functional studies and experimental validation demonstrated that SPTLC2 promoted microglial pro-inflammatory phenotype, metabolic reprogramming towards glycolysis, and exacerbated cell–cell communication alterations. SPTLC2-specific knockdown in myeloid cells using an adeno-associated virus (AAV) in our tMCAO model alleviated neurobehavioral deficits, reduced infarct volume, and improved mitochondrial function by elevating oxidative stress and mitigating mitochondrial membrane potential depolarization. Additionally, SPTLC2 was regulated by the transcription factor FLI1, and molecular docking identified potential drugs targeting SPTLC2, including Nystatin A3, Moxidectin, and Lumacaftor.

**Conclusion:**

Our study highlights SPTLC2 as a critical mediator of microglial activation and metabolic reprogramming in ischemic stroke, providing a foundation for developing novel therapeutic strategies targeting SPTLC2 to improve stroke outcomes.

**Graphical Abstract:**

Highlights

1. SPTLC2 is identified as a key driver of neuroinflammation after ischemic stroke.

2. SPTLC2 promotes pro-inflammatory microglial activation and a metabolic shift to glycolysis.

3. Targeting the FLI1-SPTLC2 regulatory axis alleviates ischemic brain injury in mice.

4. SPTLC2 represents a promising therapeutic target for improving stroke outcomes.

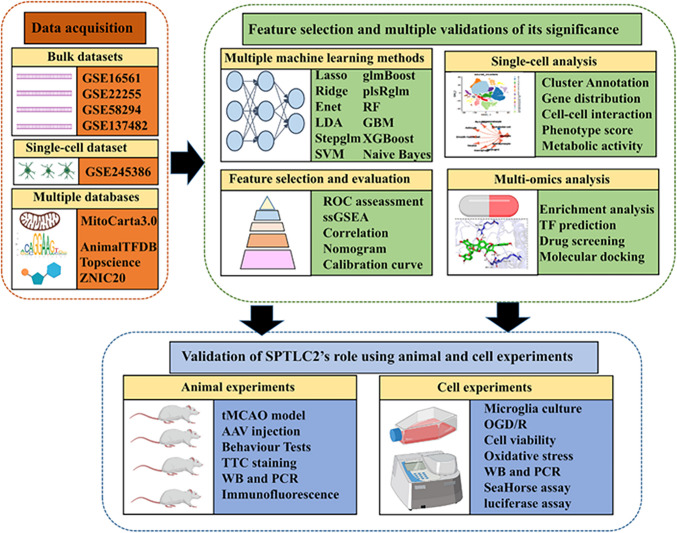

**Supplementary Information:**

The online version contains supplementary material available at 10.1007/s10565-025-10085-9.

## Introduction

Ischemic stroke (IS) is a major contributor to morbidity and mortality worldwide, and survivors frequently experience long-term disabilities (Campbell and Khatri 2020). The pathophysiology of ischemic stroke is a highly dynamic process that evolves over hours, days, and weeks. The initial acute phase is characterized by excitotoxicity and cell death, which rapidly triggers a potent neuroinflammatory response. This is followed by a subacute phase where processes of tissue repair, glial scarring, and neurogenesis are initiated, though often insufficiently. Understanding the molecular switches that regulate the transition between these phases is critical for developing therapies that can both limit initial damage and promote long-term recovery.

Mitochondria are essential organelles involved in cellular energy production and the maintenance of cellular homeostasis. During ischemia, the disruption of oxygen and glucose supply leads to a rapid depletion of adenosine triphosphate (ATP) and an increase in the production of reactive oxygen species (ROS) (Yang et al. 2018; Gao et al. 2024). This imbalance triggers a cascade of events that ultimately leads to mitochondrial dysfunction, characterized by increased inner membrane permeability, cytochrome c release, apoptotic activation, and disruption of cellular metabolism (Youle and Bliek 2012). The role of mitochondrial dysfunction in ischemic brain injury has been extensively studied over the past few decades. It is now evident that mitochondrial dysfunction exacerbates neuronal death through multiple pathways, including excitotoxicity, oxidative stress, and inflammation (Zhang et al. 2023a). Furthermore, mitochondrial dysfunction contributes to the progression of ischemic injury by promoting inflammation and edema, which further impairs cerebral blood flow and exacerbates tissue damage (Huang et al. 2023). However, the specific mechanisms through which mitochondrial dysfunction contributes to ischemic stroke pathology remain under investigation. Consequently, there is an urgent need to further explore potential therapeutic strategies targeting mitochondrial dysfunction to improve stroke outcomes.

High-throughput and single-cell RNA sequencing (RNA-seq and scRNA-seq, respectively) have transformed genomics, offering unprecedented views into the transcriptomic organization of biological systems and greatly improving our comprehension of genomic intricacy genomic complexity. These powerful techniques can uncover modified molecular patterns in many diseases, including stroke, at either the tissue-wide or single-cell resolution. For example, these methods generate exceptionally detailed data collections that enable the detection of subtle changes in gene expression patterns. These technologies reveal altered molecular signatures of various diseases at the tissue or cellular level, including stroke (Qiu et al. 2022; Zou et al. 2019). For instance, These technologies produce highly detailed datasets that allow for the identification of minor alterations in gene expression profiles. This level of sensitivity is essential for uncovering functionally disrupted genes and associated biological pathways in IS (Han et al. 2024; Liu et al. 2021). Furthermore, scRNA-seq enables the analysis of individual cells, uncovering distinct transcriptional profiles and cell types that may be overlooked when using bulk RNA-seq methods (Garcia-Bonilla et al. 2024). Additionally, these bioinformatics analysis techniques assist in discovering novel biomarkers and therapeutic targets (Shi et al. 2024). Despite the efforts of previous bioinformatics studies to explore potential biomarkers and molecular mechanisms of IS, notable limitations remain, particularly the lack of comprehensive multi-dataset analyses from both tissue and single-cell perspectives for biomarker evaluation. This includes reasonable speculations regarding regulatory pathways and the need for external experimental validation of biomarkers. In light of these shortcomings in prior research, we aim to conduct a comprehensive analysis that integrates multiple datasets to thoroughly elucidate the role and mechanisms of biomarkers through external experiments.

For this work, we gathered scRNA-seq and bulk RNA-seq datasets from an online public repository. We employed the weighted gene co-expression network analysis (WGCNA) technique to pinpoint gene collections significantly tied to IS. Subsequently, we utilized various machine learning-based ensemble methods to determine the final mitochondria-related genes (MRGs) in the test cohort. Additionally, scRNA-seq analysis was performed on GSE245386 to uncover the cell-specific expression patterns of key MRGs, leading to the selection of *Sptlc2* for further investigation. Using a range of single-cell analysis tools, we identified phenotypic and metabolic changes, communication profiles, upstream transcriptional regulators, and potential therapeutics influenced by *Sptlc2*. Finally, external experiments further validated the effects of *Sptlc2* on brain ischemia/reperfusion (I/R) injury. Our study elucidate the critical effects of *Sptlc2* on the pathophysiology of ischemic stroke, particularly in regulating microglial metabolism and inflammation.

## Methods

### Data acquisition

The scRNA-seq dataset GSE245386 (3 Sham and 2 tMCAO samples, (https://www.ncbi.nlm.nih.gov/geo/query/acc.cgi?acc=GSE245386) Ruan et al. 2023), the bulk mRNA datasets, including GSE16561 (GP570 platform, 24 Controls and 39 IS cases, (https://www.ncbi.nlm.nih.gov/geo/query/acc.cgi?acc=GSE16561) Barr et al. 2010), GSE58294 (GP570 platform, 23 Controls and 69 IS cases, (https://www.ncbi.nlm.nih.gov/geo/query/acc.cgi?acc=GSE58294) Stamova et al. 2014), GSE22255 (GP570 platform, 20 Controls and 20 IS cases, (https://www.ncbi.nlm.nih.gov/geo/query/acc.cgi?acc=GSE22255) Krug et al. 2012), and GSE137482 (GPL19057 platform, 6 Sham and 6 MCAO  mice, https://www.ncbi.nlm.nih.gov/geo/query/acc.cgi?acc=GSE137482) were downloaded from the GEO database. Mitochondria-related genes (MRGs) were acquired from the MitoCarta3.0 database.

### Bulk mRNA datasets preprocessing and analysis

Raw gene expression values from multiple datasets underwent log2 transformation and background correction. Using annotation information from the GPL570 and GPL19057 platforms, probes were subsequently mapped to corresponding gene symbols. Additionally, probes that matched multiple genes were excluded from these datasets. When multiple duplicate probes existed, the final gene expression values were determined by calculating the average expression amount. A boxplot was employed to visualize the results of the data preprocessing. The differentially expressed genes (DEGs) were determined utilizing the limma package (Ritchie et al. 2015).

### Feature identification via machine learning-based ensembles

GSE58294 served as the training dataset, while GSE16561 was used for testing. GSE22255 was selected as an external validation dataset. Genes overlapping between the WGCNA approach and the MitoCarta database were analyzed using a machine learning-based integration pipeline. This study incorporated a total of 12 machine learning models: Lasso, Ridge Regression, Enet, LDA, Stepglm, SVM, glmBoost, plsRglm, RF, GBM, XGBoost, and Naive Bayes. Based on the 10-fold cross-validation (CV), we employed a machine learning-dependent ensemble pipeline to construct an ensemble classification prediction model. This resulted in 113 unique algorithm combinations within the study following a structured process: Initially, Z-score transformation was applied to the expression profiles of both the training and testing cohorts to enhance comparability and computational efficiency. Within the comprehensive machine learning framework, four algorithms with feature selection capabilities (Lasso, RF, Stepglm, glmBoost) were first employed to reduce the gene range. Subsequently, other eight additional algorithms were employed to develop prediction models based on the identified genes derived from the initial four algorithms, yielding a total of 113 model combinations. These combinations were utilized to optimize hyperparameters and fitted the models within the 10-fold CV framework. The performance of each model was assessed by calculating the AUC scores for these cohorts, and the highest average AUC across all datasets was identified as the best-performing model. Feature genes were subsequently derived from this top-performing model.

### Immune infiltration analysis

The ssGSEA approach was applied for estimating a total of 28 immune cell subtypes infiltration levels (Lai et al. 2022). Spearman correlation was utilized to assess the strength of the correlation. Among these, there were instances of positive correlation (q-value < 0.05, r > 0), negative correlation (q-value < 0.05, r < 0), or not significant (q-value > 0.05).

### Single-cell data preprocessing and analysis

The raw gene expression matrix from GSE245386 was transformed into Seurat objects through the Seurat R package (Satija et al. 2015). Initially, genes expressed in fewer than three cells were eliminated. To ensure cell quality, we filtered out cells expressing under 200 or more than 6000 genes, along with cells containing over 10% mitochondrial transcripts. Data normalization and highly variable genes were conducted utilizing the “NormalizeData” and “FindVariableFeatures” functions in Seurat,  respectively. Dimensionality reduction was performed using PCA approach. The Harmony package was conducted to correct batch effects across multiple samples, followed by the “FindNeighbors” and “FindClusters” functions (resolution = 0.3). The “RunTSNE” function was utilized for non-linear dimensionality reduction. Finally, cell identities were assigned manually by referencing established marker genes. The “FindMarkers” function helped identify differentially expressed markers for each cluster.

### Metabolic activity analysis

The metabolic activity score for each cell across more than 80 KEGG metabolic pathways was calculated using the “AUCell” algorithm within the scMetabolism R package, employing default parameters. Additionally, we compared the metabolic scores of each group applying the Wilcoxon rank-sum test with the Bonferroni-Hochberg correlation. For visualization, we utilized the 'DotPlot.metabolism' function.

### Estimation of the scores of distinct phenotypes

The characteristic genes associated with distinct phenotypes (FERROPTOSIS, AUTOPHAGY, ACUTE INFLAMMATORY RESPONSE, and ENDOPLASMIC RETICULUM STRESS) were retrieved from the Molecular Signatures Database (MSigDB) (Liberzon et al. 2011). The M1/M2 polarization-related features were sourced from a previous study (Ma et al. 2023a). Subsequently, the “AUCell” algorithm within the AUCell package was employed to calculate phenotype-related scores for the selected cell subtypes using the default settings.

### Cell–cell‑communication analysis

The CellChat R package was utilized to comprehensively infer and assess ligand-receptor interactions among various cell types with the default parameters of recommended pipelines (Jin et al. 2021). Briefly, the initial Seurat object from each group was converted into a standard CellChat object using the “createCellChat” function, followed by embedding the CellChat database of ligand-receptor pairs into the object. Differentially expressed genes and ligand-receptor pairs were identified using the "identifyOverExpressedGenes" and "identifyOverExpressedInteractions" functions with default parameters, respectively. Subsequently, the "computeCommunProb" function was employed to infer the probability and strength of cell–cell interactions. The Sham- and tMCAO-treated groups were merged using the "mergeCellChat" function. The total amount of communications and interaction strength were compared utilizing the "compareInteractions" function, and the " netVisual_circle” and netVisual_bubble" functions were utilized to display the ligand-receptor strength for merged objects.

### Molecular docking

Molecular docking, a computational method for investigating receptor-ligand interactions, was performed to assess the binding affinities of candidate compounds to their targets (Zhang et al. 2023b). The SPTLC2 protein (3D structure) was acquired from the AlphaFold Protein Structure website. Ligand structures in SDF format were sourced from the Topscience database, while FDA-approved drug compounds were retrieved from the ZINC20 database. The ligands with SDF format were transformed into PDBQT format using the OpenBabel software for subsequent docking analysis. Additionally, the PyMOL software was employed to delete water molecules and ligands of SPTLC2 protein. These cleaned proteins were then hydrogenated, docked, and converted into PDBQT format using AutoDock Vina software. The binding site parameters, including X, Y, Z coordinates, were predicted using the PrankWeb website. Finally, molecular docking was conducted utilizing Uni-Dock software.

#### Animal model and behavioral testing

An in vivo model of ischemic stroke was established using transient middle cerebral artery occlusion (tMCAO) in male C57BL/6J mice. All animal procedures were conducted in accordance with approved ethical guidelines. A comprehensive description of the animal housing, surgical protocols, post-operative care and infarct volume measurement are available in the supplemental materials and methods.

### Adeno-associated virus (AAV) injection

Myeloid cells-specific adeno-associated virus (AAV) bearing the Iba1 promoter (AAV2/9-Iba1-mir30-sh*Sptlc2*) was constructed by Hanheng Biotech (Shanghai, China). A total of 3μLof AAV was stereotactically injected into the cortex (titer:1.8 × 10^12^ GC/mL), with the injection coordinates as follows: AP = 0.2, ML = 3.0, and DV = 1.5. The injection rate was set to 100 nL/min. The syringe was retained for more than 15 min to prevent reflux of the virus, after which it was withdrawn slowly. Following injection, mice were allowed a three-week recovery period to ensure stable and maximal transgene expression. After this period, the mice were subjected to the tMCAO procedure, followed by behavioral and histological analyses at the indicated time points.

### Behavior tests

All behavioural tests were conducted between 9:00 AM and 4:00 PM, during the light phase of the circadian cycle, to minimize variability. Neurological function in mice was evaluated at multiple time points following tMCAO based on the modified Neurological Severity Score (mNSS), rotarod test, novel object recognition (NOR) test, and adhesive removal test. Prior to surgery, all animals underwent a three-day pretraining period. (1) mNSS score: The mNSS is a composite scale used to evaluate neurological function after cerebral ischemia. It assesses motor (muscle tone, movement abnormalities), sensory (visual, tactile, proprioceptive), reflex, and balance functions. The scoring ranges from 0 (no deficit) to 18 (maximum impairment), with higher values indicating more severe neurological damage. Specific tasks include lifting the mouse by the tail (to assess motor and reflex responses), placing it on the floor (to evaluate motor and sensory abilities), and beam balance and reflex tests. A higher score correlates with greater functional impairment. (2) Rotarod test. This assessment gauges motor coordination and equilibrium (Sun et al. 2023a). In the training stage, mice were set on a spinning rod moving at a steady 5 rpm for five minutes. If a mouse fall, it was put back on the rod to restart the trial. This training was repeated for three straight days. On the fourth day, we used an accelerating protocol, which began at 5 rpm and ramped up to 40 rpm over a five-minute period.  The latency to fall was recorded. Three repetitions of this test were done per day for each animal, separated by a 15-min rest. (3) NOR test. The NOR assessment was employed to measure cognitive abilities, based on a previously reported procedure (Toriumi et al. 2021). For habituation, mice could freely investigate an open field chamber for ten minutes daily for three days in a row. On the fourth day, two matching objects were placed in the arena, and each animal was allowed to explore them for 10 min. The interaction time with each object was tallied. On the fifth day, one object was substituted with a new one, and exploration time was recorded for five minutes. We calculated a recognition index (RI) to measure recognition memory, defined as the proportion of time investigating the new object over the total time spent exploring both objects. (4) Adhesive removal test. This procedure evaluates somatosensory and motor capacities (Hagberg et al. 2012). A small piece of tape was placed on either the forepaw on the same side as the injury (ipsilateral) or the opposite side (contralateral).  The time required for the mouse to remove the adhesive from each paw was recorded as a measure of sensorimotor function . Before surgery, mice were pretrained for three days to confirm they had no pre-existing sensorimotor impairments. The test was halted if a mouse took longer than the 120-s maximum time to remove the tape. Every mouse performed this test three times.

### Primary microglia culture

We isolated primary microglia from the cerebral cortices of C57BL/6J mouse pups aged 0 to 4 days (P0-P4), using established protocols with minor adjustments (Plastira et al. 2020). Briefly, we dissected cerebral cortices from whole brains, removed the meninges, and digested the cerebral cortices with 0.1% trypsin for 20 min at 37 °C in a 5% CO₂ environment.

Then, DMEM with 10% fetal bovine serum was added to halt the digestion. The cell suspension was centrifuged at 800 g for ten minutes at 37 °C, and the resulting pellet was resuspended and plated in 75 cm^2^ flasks coated with poly-D-lysine (PDL). After a 10- to 14-day incubation period, we separated microglia from the mixed glial bed by shaking the flask manually 10–20 times. We collected the detached microglial cells and seeded them into PDL-coated flasks or plates for later experiments.

### Construction of oxygen–glucose deprivation/reoxygenation (OGD/R) model

The OGD/R model was created to mimic ischemia/reperfusion damage in cultured microglial cells. First, microglia were placed in glucose-free DMEM (Gibco, NY, USA) to   deplete residual glucose. Then, the cells were moved into an anaerobic incubator with a gas mix of 5% CO₂ and 95% N₂ at 37 °C for two hours, which induced oxygen and glucose deprivation. Afterward, we replaced the medium with standard growth  medium and put the cells back in a normal oxygen incubator at 37 °C with 5% CO₂ for various durations to trigger reoxygenation damage.

### Cytotoxicity assay

Cell viability was quantified using a Lactate Dehydrogenase (LDH) Assay Kit (Beyotime, Jiangsu, China). We treated microglial cells with 0.1% trypsin (Gibco, New York, USA) and then centrifuged them at 800 rpm for ten minutes. The collected supernatant was moved to a 96-well plate and mixed with the LDH reaction solution. A microplate reader measured the absorbance at a wavelength of 490 nm. We calculated cell viability as the percentage of LDH released compared to the total cellular LDH amount.

### Transcription factor prediction and luciferase assay

Four web tools, namely hTFTarget, ENCODE, CHIP_Atlas and KonckTF, were utilized to determine the upstream transcription factor regulating SPTLC2. The AnimalTFDB web tool was also used to forecast potential transcription factor binding sites (TFBS). Prior to the luciferase reporter assay, we amplified the coding sequence of murine *Fli1* and inserted it into the pcDNA3.1(+) vector to create the pcDNA3.1-*Fli1* overexpression plasmid. We also amplified the promoter region of the mouse *Sptlc2* gene and cloned it into the pGL3-basic firefly luciferase reporter vector, which yielded the wild-type reporter construct pGL3-*Sptlc2*-WT. In parallel, we performed site-directed mutagenesis on the *Sptlc2* promoter region within the pGL3-basic vector to create the mutant plasmid pGL3-*Sptlc2*-Mut. For the dual-luciferase reporter experiment, we cultured BV2 cells in 24-well plates and transfected them with Lipofectamine 3000 (Invitrogen, MA, USA), according to the product's protocol. We measured both firefly and Renilla luciferase activities 48 h after transfection with a dual-luciferase assay system (Promega, Madison, WI, USA). The relative luciferase activity was determined by normalizing the firefly luciferase reading to the Renilla luciferase reading.

Weighted Correlation Network Analysis (WGCNA), Functional enrichment analysis, Transfection with Lentivirus, Quantitative real-time polymerase chain reaction (qRT-PCR), Western-Blot, Assessment of ROS generation, Mitochondrial Membrane Potential (MMP) Assay, SeaHorse assay, and Immunofluorescence are described in detail in supplemental materials and methods.

### Statistical analyses

All statistical evaluations were completed using R software (version 4.3.2) and GraphPad Prism (version 8.0). The data are shown as the mean ± standard deviation (SD) and were obtained from a minimum of three separate experiments. We used the Shapiro–Wilk test to check for data normality and Levene's test to check for the homogeneity of variances. To compare two groups, we employed the Student’s t-test for data that was normally distributed, and the Wilcoxon rank-sum test for data that was not. For comparisons of three or more groups, we used a one- or two-way analysis of variance (ANOVA), followed by a Bonferroni post hoc test. If the data were not normally distributed, we used the Kruskal–Wallis test, followed by Dunn’s multiple comparisons test. Behavioral data gathered over multiple time points were examined with a two-way repeated-measures ANOVA.  A p-value <0.05 was considered statistically significant.

## Results

### Identified two critical modules strongly correlated with IS using WGCNA

**Fig. 1:**
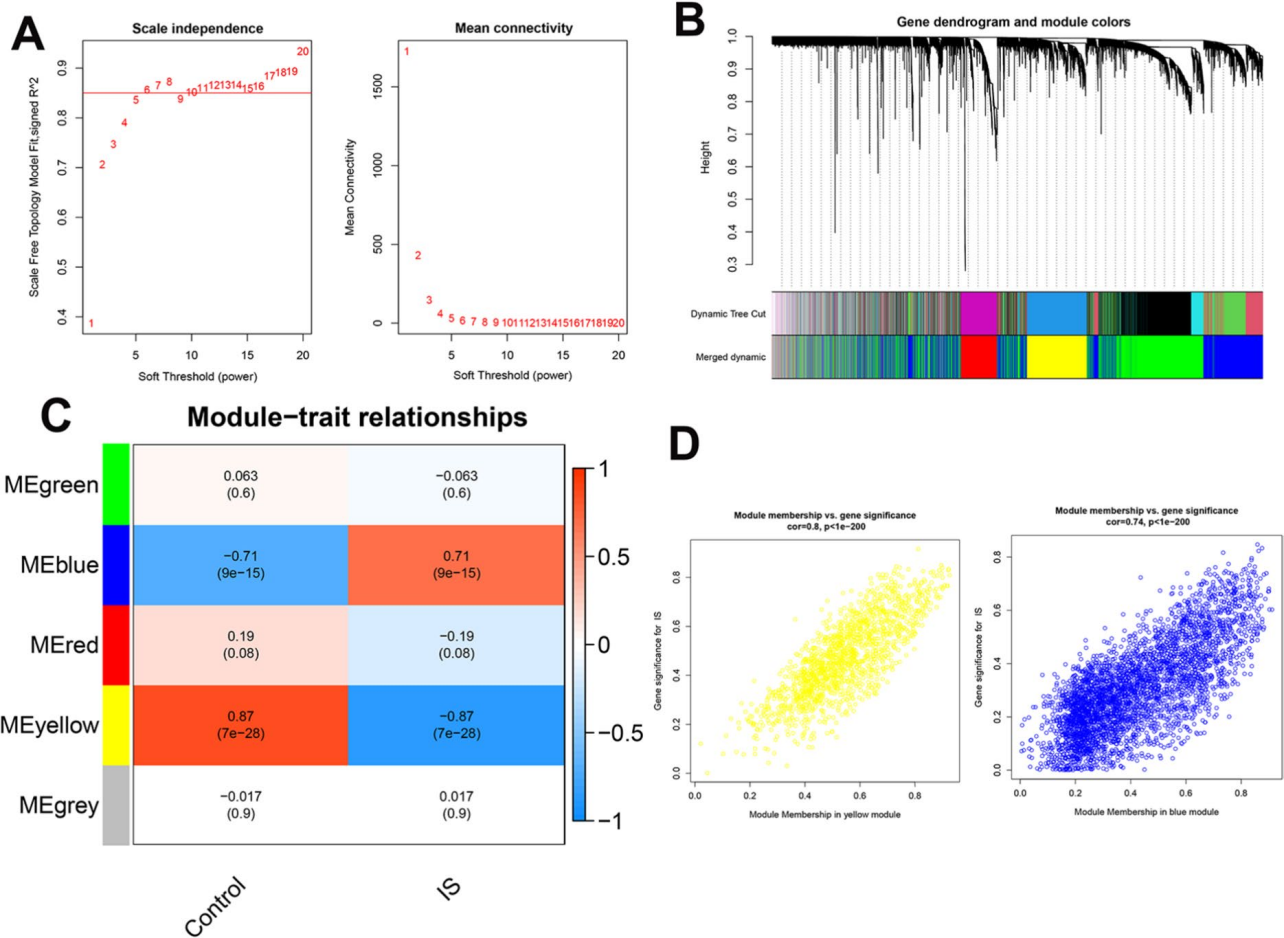
WGCNA-based identification of crucial modules linked to IS. (**A**) Analysis showing the selection of the ideal soft-threshold power alongside mean network connectivity. (**B**) A dendrogram of genes where unique colors represent the different modules discovered through WGCNA. (**C**) A correlation matrix presented as a heatmap, illustrating the relationship of gene modules to clinical condition (IS versus Control). (**D**) A graph plotting Gene Significance (GS) against Module Membership (MM), demonstrating a strong positive relationship in the yellow and blue modules

To map the gene expression network in IS, we applied WGCNA to generate a co-expression gene network using the expression data of the top 50% most variable genes from GSE58294. A soft threshold of 6 was chosen as optimal, which ensured the network satisfied the scale-free topology condition (Fig. 1A). A topological overlap matrix (TOM) was then created using this threshold, and this matrix was used for dynamic tree clustering of genes. This process identified gene modules with a significant link to IS (Fig. 1B). We then assessed the correlation between these modules and the clinical  status (IS vs. control). This revealed that the yellow and blue modules strongly correlated with IS (Fig. 1C, R > 0.7 and p < 0.05). Intra-module analysis showed a high correlation between module membership (MM) and gene significance (GS) for both the yellow module (R = 0.8, p < 0.001) and the blue module (R = 0.74, p < 0.001) (Fig. 1D). A total of 5348 genes (1533 in the yellow module, 3815 in the blue module) related to IS were selected for further investigation. We then performed an enrichment analysis to  determine whether these two modules captured the biological meaning of IS. GO enrichment results suggested that the genes were mainly involved in immune functions, nerve regeneration, transport across the plasma membrane, cytokine synthesis, and chemokine receptor functions (Figure S1A). KEGG analysis showed these genes were mostly participated in signaling molecules and their interactions, the immune system, signal transduction pathways, and lipid metabolism (Figure S1B). Taken together, these findings suggest that the identified genes are central to regulating a wide range of biological activities related to IS.

### Machine learning-based integration identified mitochondrial-related features of IS

**Fig. 2:**
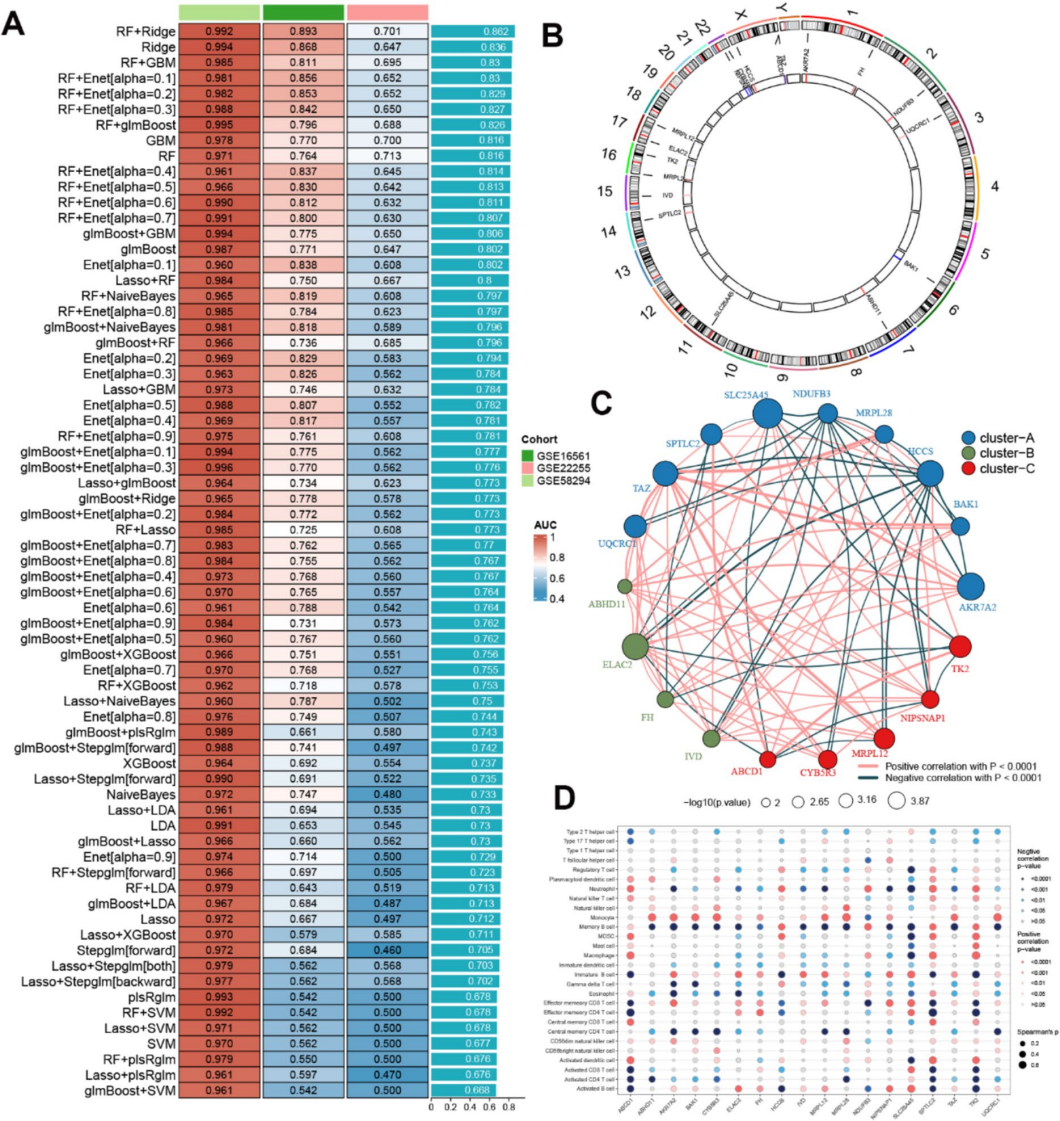
Pinpointing essential MRGs via an integrated machine learning strateg. (**A**) A feature selection process utilized 113 predictive models applied to 155 common MRGs across training, testing, and validation sets. The AUC for every model was calculated, and the 70 models with the best mean AUCs are shown. (**B**) A display showing the chromosomal locations for the discovered MRGs. (**C**) A network diagram illustrating connections between the 18 identified MRGs. The circle's diameter corresponds to the gene's significance in IS. For visualization, three separate gene groups are colored blue, green, and orange. (**D**) A matrix showing the relationship between 18 key MRGs and 28 types of immune cells. Circle size indicates the correlation strength; red denotes a positive relationship, whereas dark green shows a negative one

To further identify mitochondrial-related signature genes (MRGs), we first crossed the two primary gene modules derived from WCGNA with genes obtained from the Mitocartd 3.0 database, resulting in the identification of 155 overlapping MRGs (Fig. S2). Subsequently, the expression profiles of these genes underwent a machine learning-based integration process to distinguish the key MRGs associated with IS. A total of 113 predictive classification models were conducted to fit the training (GSE58294), testing (GSE16561), and validation (GSE22255) cohorts utilizing the CV framework. The combination of RF and Ridge regression models displayed the highest average AUC score (0.865) among all datasets, establishing it as the optimal classification model (Fig. 2A). Consequently, a total of 18 MRGs (ABCD1, ABHD11, AKR7A2, BAK1, CYB5R3, ELAC2, FH, HCCS, IVD, MRPL12, MRPL28, NDUFB3, NIPSNAP1, SLC25A45, SPTLC2, TAZ, TK2, and UQCRC1) were identified based on the above model (Fig. 2A). The specific positions of these MRGs on the chromosome are depicted in Fig. 2B. To investigate the correlation among the identified MRGs, we constructed a gene–gene interaction network based on their expression profiles, which revealed three distinct clustering patterns, Notably, complex interactions were observed among these 18 MRGs, suggesting potential synergistic or antagonistic effects under IS (Fig. 2C). Furthermore, we examined the association between these 18 MRGs and 28 immune cell subsets. We observed positive correlations between most  MRGS  and multiple immune cell subsets, including effector memory CD8 + T cells, monocytes, immature B cells, and natural killer cells (p-value < 0.05) (Fig. 2D), revealing the critical roles of MRGs in modulating immune-related pathways.

### Developing and validation of candidate MRGs of IS

**Fig. 3:**
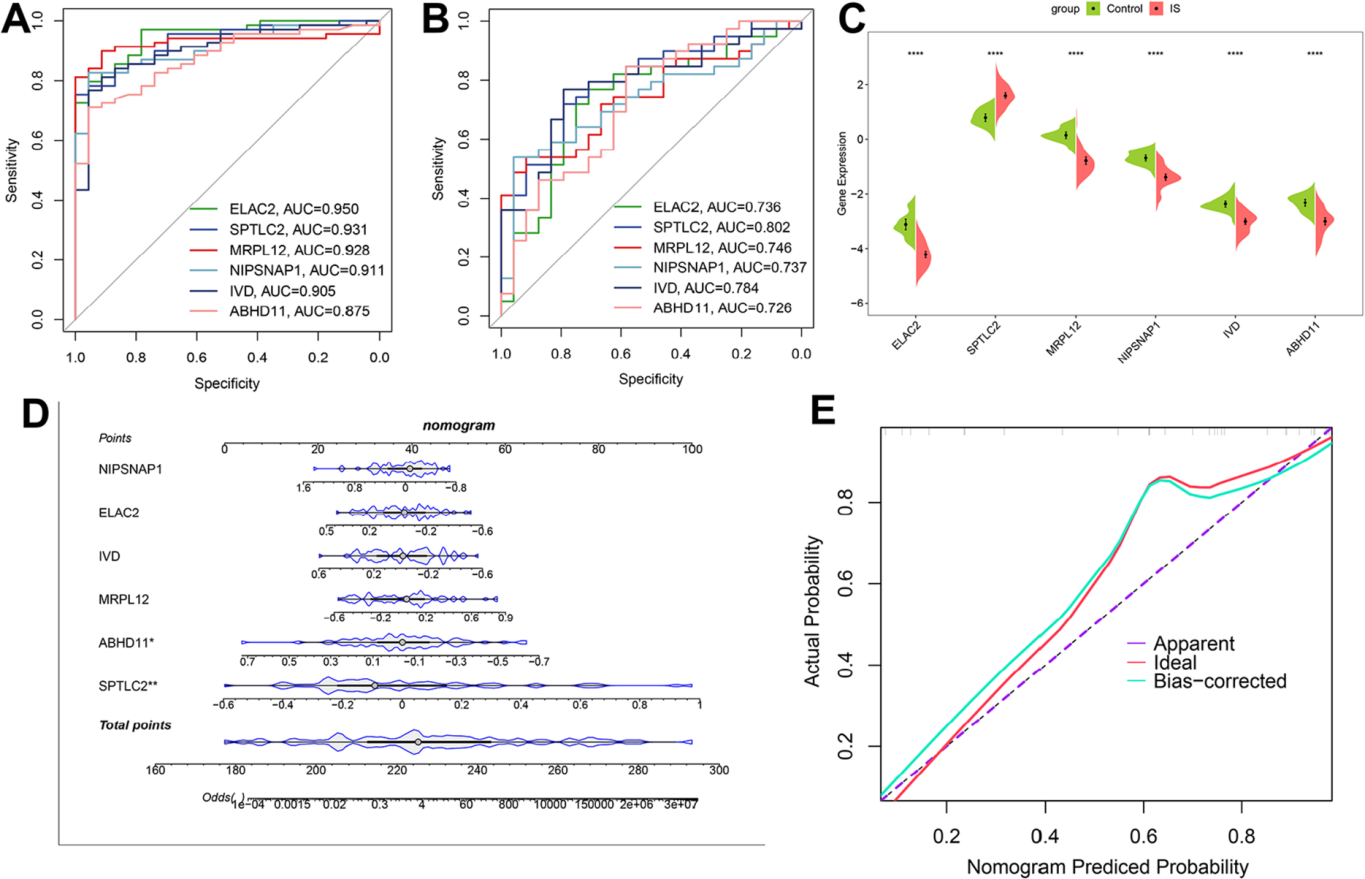
Corroboration of MRG significance and creation of a predictive nomogram. (**A**, **B**) Receiver operating characteristic (ROC) curve assessments for the six central MRGs using the training set (**A**) and the test set (**B**). (**C**) Confirmation of the expression patterns of these six MRGs within the IS testing group. (**D**) A nomogram developed from the six primary MRGs. (**E**) A calibration plot evaluating the accuracy of the nomogram in the testing group. Wilcoxon rank-sum test indicates ^****^p < 0.0001 relative to the Control group

To estimate the diagnostic ability of these 18 MRGs, we further performed the ROC curve analysis utilizing the GSE58294 and GSE16561 datasets. The results indicated that the AUC scores for ELAC2, SPTLC2, MRPL12, NIPSNAP1, IVD, and ABHD11 surpassed 0.87 and 0.72 in the GSE58294 (0.950 for ELAC2, 0.931 for SPTLC2, 0.928 for MRPL12, 0.911 for NIPSNAP1, 0.905 for IVD, and 0.875 for ABHD11) and GSE16561 cohort (0.736 for ELAC2, 0.802 for SPTLC2, 0.746 for MRPL12, 0.737 for NIPSNAP1, 0.784 for IVD, and 0.726 for ABHD11), respectively (Fig. 3A-B), demonstrating their high sensitivity and specificity in distinguishing IS from control. Furthermore, the expression differences among these six MRGs were verified, revealing that only SPTLC2 exhibited increased expression during the development of IS (Fig. 3C). Additionally, a nomogram model for IS diagnosis was constructed utilizing these 6 MRGs (ELAC2, SPTLC2, MRPL12, NIPSNAP1, IVD, and ABHD11) (Fig. 3D). The nomogram model’s predictive ability was then evaluated with a calibration curve, exhibiting minimal error between the actual and predicted risk of IS. Figure 3E illustrates the performance of the nomogram model, highlighting its high accuracy in predicting IS. Therefore, these 6 MRGs were identified as critical features that could serve as potential diagnostic biomarkers for IS.

### scRNA-seq analysis elucidated the diverse cell-specific expression patterns of characteristic MRGs in IS

**Fig. 4:**
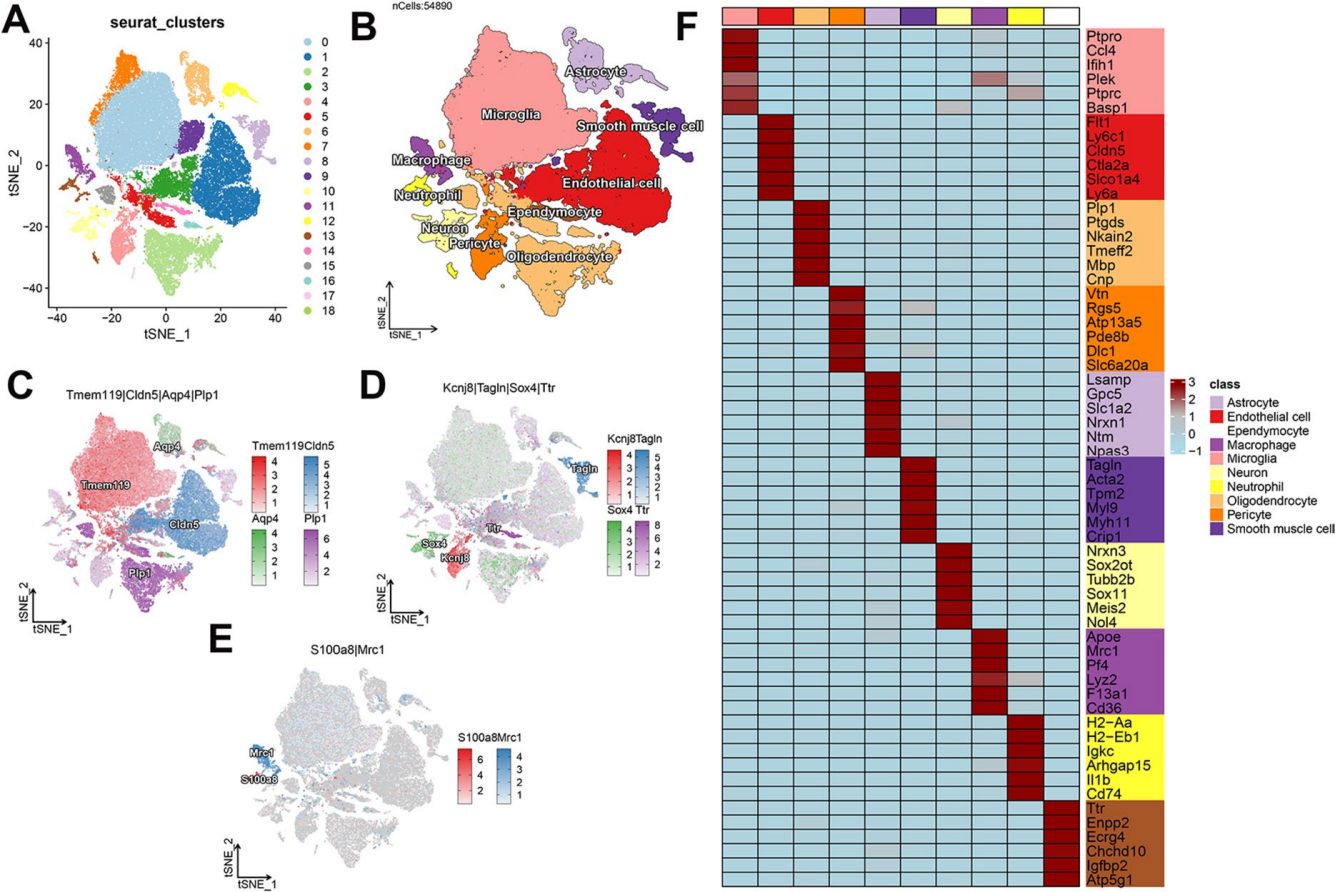
Delineation and annotation of major cellular clusters through scRNA-seq. (**A**) Based on a tSNE plot, a total of 19 distinct clusters were visualized with different colors. (**B**) Using marker gene expression, each cluster was annotated, resulting in the final determination of 10 cell types. (**C**-**E**) tSNE visualizations demonstrating the gene expression for markers specific to certain clusters. (**F**) A heatmap displaying the top six marker genes for every cell type. Expression values for each gene were log-normalized and then scaled (Z-score) across all cells. The color gradient, from blue (low) to brown (high), indicates the scaled expression value

To clarify the expression patterns of these 6 key genes across different cell types in IS, we first utilized scRNA-seq analysis of GSE245386 to quantify the major cell subtypes present in IS. A total of 54890 high-quality cells from 5 samples were identified as suitable for further study. Subsequently, unsupervised clustering yielded 19 distinct clusters (Fig. 4A). These clusters were further characterized as Astrocyte, Endothelial cell, Ependymocyte, Macrophage, Microglia, Neuron, Neutrophil, Oligodendrocyte, Pericyte, and Smooth muscle cell based on classical cellular markers (Astrocyte: *Aqp4*, Endothelial cell: *Cldn5*, Ependymocyte: *Ttr*, Macrophage: *Mrc1*, Microglia: *Tmem119*, Neuron: *Sox4*, Neutrophil: *S100a8*, Oligodendrocyte: *Plp1*, Pericyte: *Kcnj8*, Smooth muscle cell: *Tagln*) (Fig. 4B-E). The top 6 differentially  expressed genes among the ten cell types are presented in Fig. 4F.

**Fig. 5:**
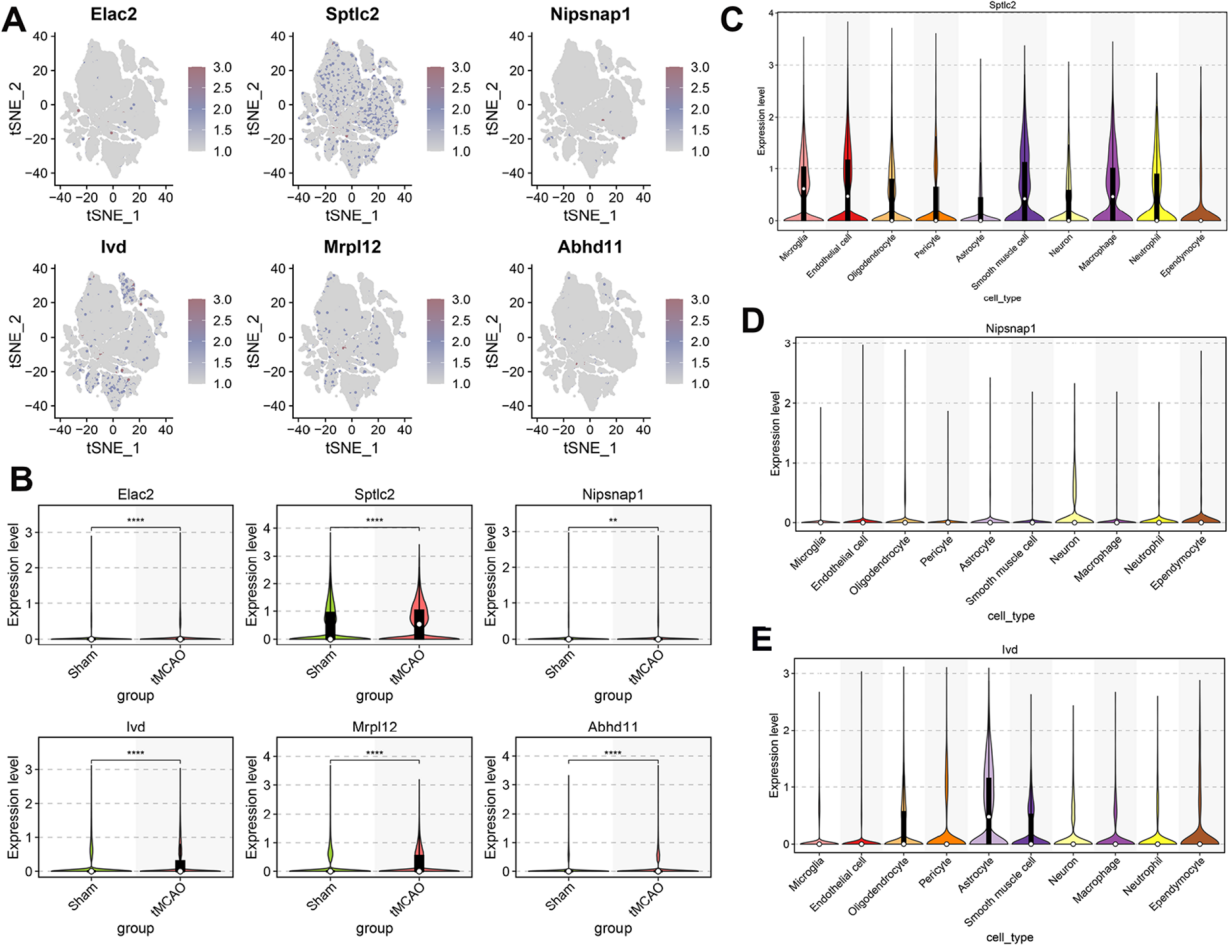
scRNA-seq reveals diverse cell-specific expression of six key MRGs during cerebral I/R. (**A**) A tSNE visualization mapping the expression of the six pivotal MRGs and their proportional representation across different cell populations. (**B**) A violin plot contrasting the expression of these six MRGs between the Sham and tMCAO cohorts. (C-E) Violin plots detailing the expression variability of *Sptlc2* (C), *Nipsnap1* (**D**), and *Ivd* (E) across the different cell types. The Wilcoxon rank-sum test showed ^****^p < 0.0001 compared to the Sham group

Next, the cell-specific expression patterns of the 6 MRGs were further explored (Fig. 5A). We found *Sptlc2* was prominently expressed in microglia, endothelial cells, smooth muscle cells, macrophages, and neutrophils. Additionally, Nipsnap1 and Ivd exhibited predominant expression in neurons and astrocytes, respectively. Notably, the expression of *Sptlc2* in the IS group was significantly higher than that in the control group, corroborating the findings from bulk transcriptome analysis (Fig. 5D-E). In contrast, no significant differences across all cell types were observed in *Elac2*, *Mrpl12*, and *Abhd11* (Figure S3). Therefore, *Sptlc2* was selected for further investigation.

### SPTLC2 expression fluctuates in a time-dependent manner after cerebral I/R injury

**Fig. 6:**
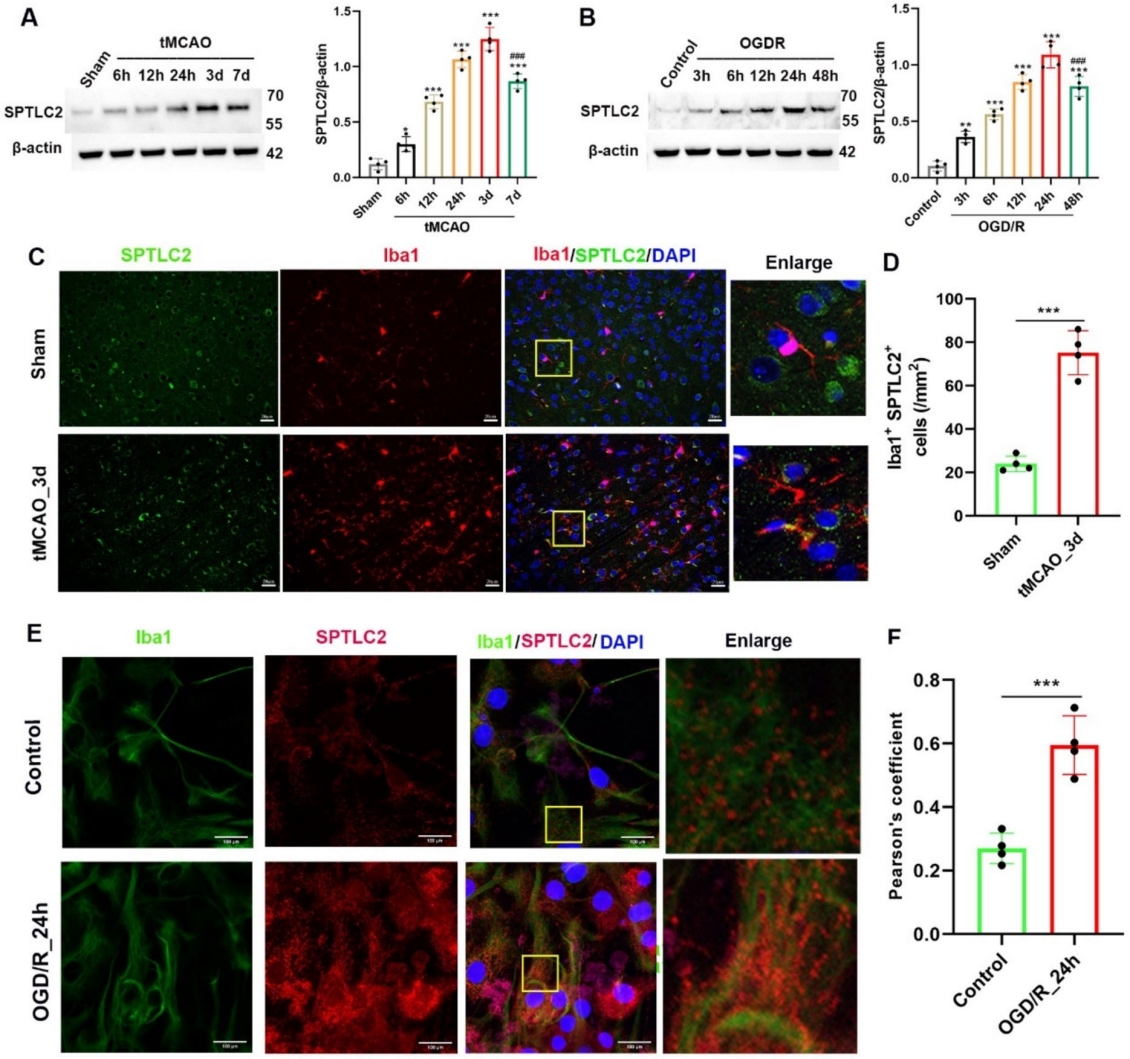
SPTLC2 expression is upregulated in response to cerebral I/R injury. (**A**) Western blot analysis and corresponding quantification of SPTLC2 protein in peri-infarct cortical regions from tMCAO-afflicted mice. (**B**) Protein analysis and quantification for SPTLC2 in primary microglial cells subjected to OGD/R damage. Data are presented as mean ± SD. ^*^p < 0.05, ^**^p < 0.01, ^***^p < 0.001 versus the Sham or Control groups; ^###^*p* < 0.001 versus the tMCAO_3d or OGD/R_24h groups, determined by one-way ANOVA. (**C**) Representative immunofluorescence images showing microglia (Iba1) and SPTLC2 in the peri-infarct cortex three days post-tMCAO. Scale bar represents 20 µm. (**D**) Quantitative analysis of co-localized cells following the tMCAO procedure. (**E**) Immunofluorescence showing SPTLC2 in cultured primary microglia after OGD/R insult. Scale bar signifies 100 µm. (**F**) Pearson correlation quantification between primary microglia (Iba1) and SPTLC2. Green = SPTLC2; red = Iba1; blue = DAPI. n = 4 per group. Values are shown as mean ± SD. ^***^p < 0.001 compared to Sham mice or Control microglia, assessed by Student’s t-test

We conducted a series of in vivo and in vitro experiments to verify the SPTLC2 functions in cerebral ischemia/reperfusion injury. with the detailed procedure illustrated in Figure S4. We first conducted  Western Blot to evaluated the dynamic changes in SPTLC2 expression in samples from the penumbral region of sham-operated and tMCAO mice. We found the protein and mRNA levels of SPTLC2 increased continuously at 6, 12, 24 h and 3 days post-reperfusion, but declined during the later stage of cerebral ischemia–reperfusion injury (days 7) (Fig. 6A). Relative fluorescence intensity analysis revealed that SPTLC2 expression was predominantly localized in microglia, showing a significant increase at 3 days after reperfusion compared to sham-operated mice (Fig. 6C). Correspondingly, SPTLC2 protein levels in primary microglia peaked at 24 h and gradually decreased over day 2 following OGD/R injury (Fig. 6B). Furthermore, SPTLC2 immunostaining signals were upregulated in primary microglia after OGD/R injury (Fig. 6D). These findings suggest that SPTLC2 is upregulated in the ischemic penumbra, and the dysregulated expression of SPTLC2 in microglia may cause brain I/R injury.

### SPTLC2 suppression alleviates the impairment of neurobehavioral function after cerebral I/R injury

**Fig. 7:**
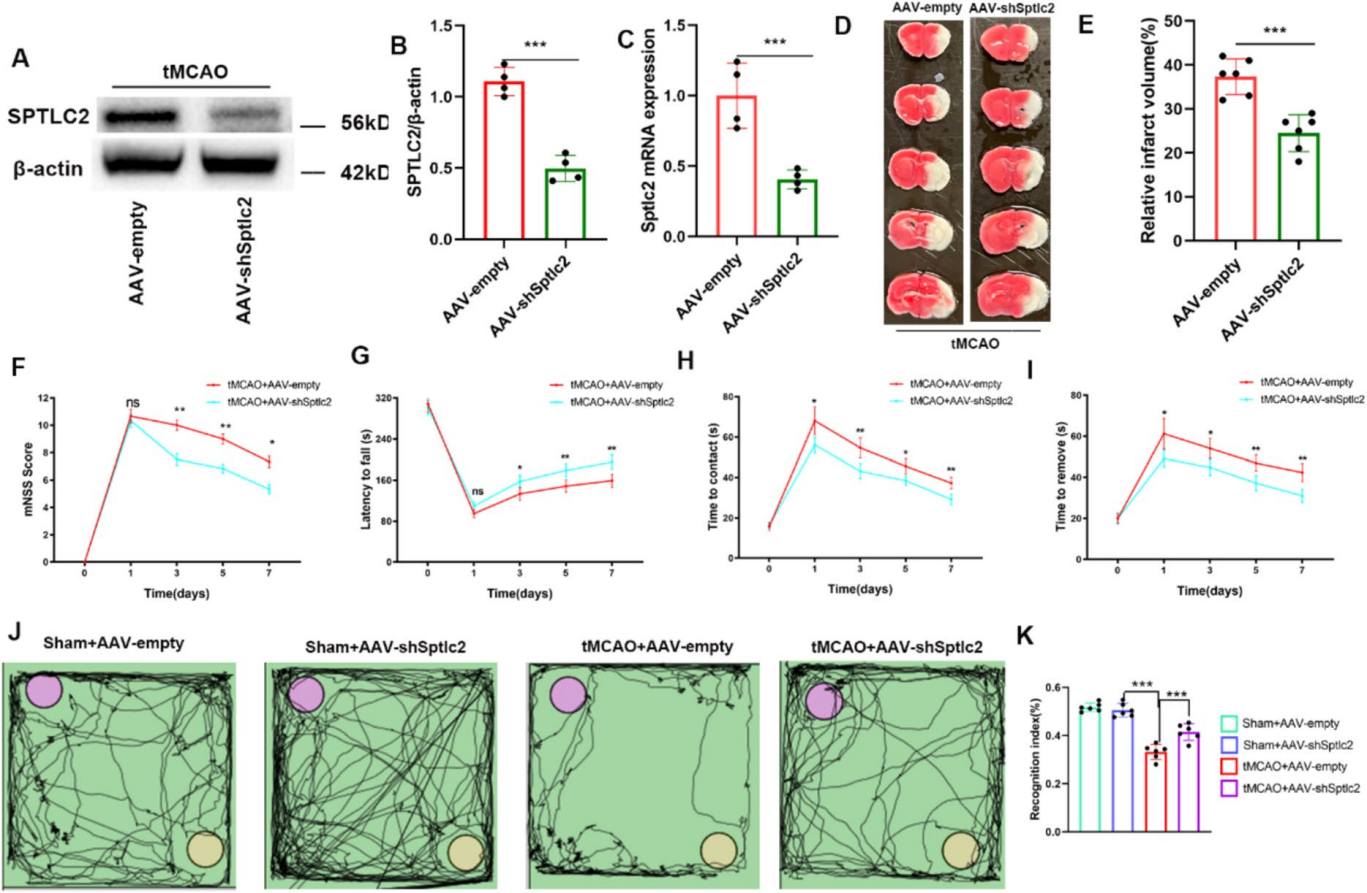
Neurological damage following cerebral I/R is ameliorated by SPTLC2 knockdown. (**A**-**C**) Assessment and quantification of SPTLC2 knockdown efficacy using Western blotting and RT-PCR; *n* = 4 per group. Student’s t-test indicates ^***^p < 0.001 versus the tMCAO + AAV-empty group. (**D**, **E**) Representative images from TTC staining (**D**) and corresponding measurement (**E**) of cerebral infarct volume across different cohorts; *n* = 6 per group. Student’s t-test shows ^***^p < 0.001 versus the tMCAO + AAV-empty group. (**F**-**I**) Sensorimotor deficits were assessed at multiple time points using the mNSS score (**F**), rotarod performance (**G**), and adhesive removal task (H, I); n = 6 per group. Two-way repeated measures ANOVA shows ^*^P < 0.05, ^**^p < 0.01 compared to the tMCAO + AAV-empty group. (**J**) The novel object recognition (NOR) test was used at various time points to assess cognitive ability. A purple circle denotes a familiar object, while a yellow circle indicates a novel one. (**K**) Quantitative measurement of the recognition index; *n* = 6 per group. One-way ANOVA indicates ^***^p < 0.001 versus the Sham + AAV-sh*Sptlc2* or tMCAO + AAV-empty groups. All values are expressed as mean ± SD

To investigate the impact of SPTLC2 knockdown on ischemia–reperfusion brain injury, mice were injected with myeloid cells-specific SPTLC2 knockdown with adeno-associated virus (AAV2/9-Iba1-mir30-sh*Sptlc2*) or negative control (AAV-empty) and then subjected to tMCAO injury. We confirmed a remarkable reduction in SPTLC2 expression at the protein and mRNA level in the AAV-sh*Sptlc2* group (Fig. 7A-C and Figure S5). Notably, SPTLC2 expression increased significantly on day 3 post-I/R, therefore, day 3 was selected as the time point for detection following ischemic injury. As depicted in Fig. 7D-E, TTC staining of brain sections revealed that the infarct volume was significantly reduced in SPTLC2 knockdown mice after tMCAO. We subsequently assessed the effects of SPTLC2 on sensorimotor and motor outcomes through mNSS, rotarod test, and adhesive removal test. The mNSS score significantly decreased from days 1 to 7 following I/R injury, while SPTLC2 knockdown partially mitigated this decline, restoring scores on day 3 post-injury (Fig. 7F). Furthermore, compared to the tMCAO group, SPTLC2 knockdown mice demonstrated significantly longer durations on the rotarod, indicating enhanced motor performance and balance (Fig. 7G). Consistent findings were observed in adhesive removal test evaluating sensorimotor function in tMCAO mice, with SPTLC2 knockdown mice exhibiting shorter contact and removal times three days after reperfusion injury compared to those subjected to I/R injury (Fig. 7H-I). Furthermore, SPTLC2 knockdown significantly alleviated the impairment of recognition memory, as evidenced by a remarkable increase in the recognition index when compared with the tMCAO group (Fig. 7J-K). Collectively, these findings reveal that the reduction of SPTLC2 may restore neurological function following cerebral ischemia–reperfusion injury.

### SPTLC2 promotes the alteration of phenotypes after cerebral I/R injury

**Fig. 8:**
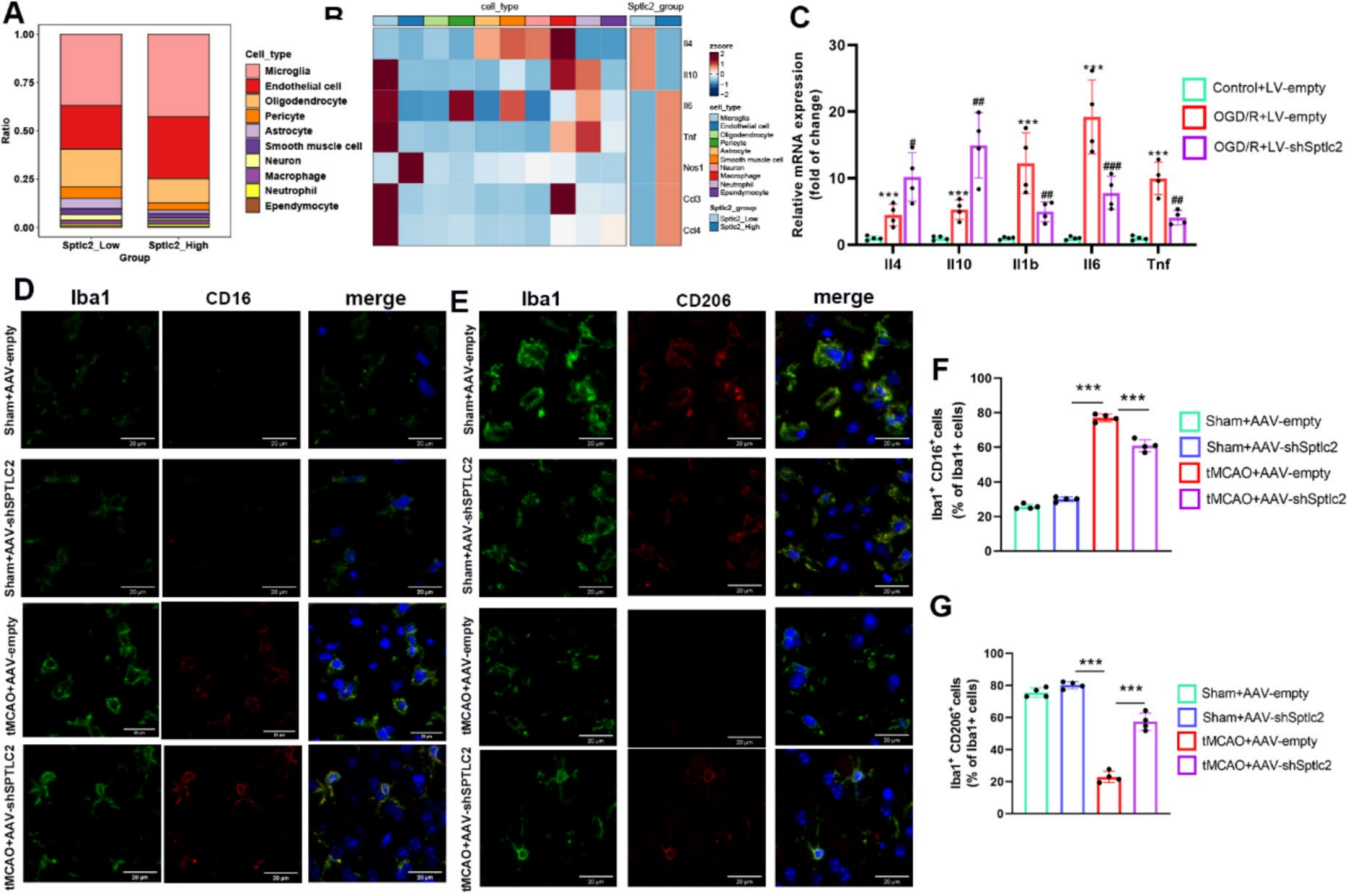
SPTLC2 knockdown modulates microglial phenotype polarization after cerebral I/R. (**A**) A bar chart showing the relative percentage of cell populations in the *Sptlc2*_Low and *Sptlc2*_High cohorts. The sum proportion for each cell type is 100% within each group. (**B**) A heatmap illustrating expression differences for pro- and anti-inflammatory genes among cell types or between high and low SPTLC2 expression groups. (**C**) RT-PCR quantification of pro- and anti-inflammatory markers between groups; n = 4 per group. Data are presented as mean ± SD. One-way ANOVA shows ^***^p < 0.001 vs. Control + LV-empty group, and ^#^*p* < 0.05, ^##^*p* < 0.01, ^###^*p* < 0.001 vs. OGD/R + LV-empty group. (**D**, **E**) Representative fluorescence images of Iba1 (green)/CD16 (red)/DAPI (blue) and Iba1 (green)/CD206 (red)/DAPI (blue) in the peri-infarct region. Scale bar: 20 μm. (**F**, **G**) Quantitative results for the proportion of CD16 (**F**) and CD206 (**G**) positive cells; n = 4 per group. Data are shown as mean ± SD. One-way ANOVA indicates ^***^p < 0.001 vs. the Sham + AAV-sh*Sptlc2* or tMCAO + AAV-empty groups

To further investigate the functions mediated by SPTLC2, we established a threshold of 75% for SPTLC2 expression in single cells from tMCAO mice, based on quartile analysis. This allowed us to classify the cells into two groups: *Sptlc2*_Low and *Sptlc2*_High. We observed a reduction in the proportion of both microglia and endothelial cells in the *Sptlc2*_High group (Fig. 8A). Consequently, we isolated microglia exhibiting high and low SPTLC2 expression levels, respectively, and calculated classical phenotype scores for comparison between the groups. As anticipated, *Sptlc2*_High microglia displayed significantly elevated ferroptosis-related scores, indicating that abnormal iron accumulation may result from an imbalance in iron metabolism, which subsequently induces microglial endoplasmic reticulum stress and cell death, contributing to stroke progression. Furthermore, we found significantly increased endoplasmic reticulum stress and autophagy activity in *Sptlc2*_High microglia, thereby confirming our hypothesis (Figure S6). Notably, microglia with high SPTLC2 expression exhibited enhanced pro-inflammatory phenotype scores while demonstrating reduced anti-inflammatory activity compared to those with low SPTLC2 expression. *Sptlc2*_High microglia exhibited increased pro-inflammatory factors (Il6, Tnf, Nos1, Ccl3, and Ccl4) expression and reduced activity of anti-inflammatory factors (Il4 and Il10) (Fig. 8B). In addition, we found tMCAO injury could increase the number of Iba1 + microglia. Importantly, SPTLC2 knockdown significantly attenuated this stroke-induced increase in total microglial numbers (Figure S7). To further validate the relationship between SPTLC2 and microglial phenotype in vitro, we evaluated the expression of corresponding mRNA encoding pro-inflammatory genes (Il1b, Il6 and Tnf) and reparative microglia-related genes (Il4 and Il10). The OGD/R-induced microglial activation was closely related with the overexpression of pro-inflammatory factors, which could be inhibited after the treatment of SPTLC2 knockdown (Fig. 8C). Subsequently, we assessed the pro-inflammatory (CD16) and anti-inflammatory (CD206) microglia markers in the peri-infarct cerebral cortex using immunofluorescence. Following tMCAO injury, an increased proportion of CD16-positive microglia was observed in the ischemic penumbra region, a trend that was reversed by the inhibition of SPTLC2. Conversely, the downregulated proportion of CD206 was found to increase in tMCAO mice after the injection of the SPTLC2 shRNA (Fig. 8D-G). These findings suggest that SPTLC2 may facilitate the transition of microglia to a pro-inflammatory state, thereby exacerbating I/R brain injury.

### SPTLC2 enhances the alteration of microglial metabolic states after cerebral I/R injury

**Fig. 9:**
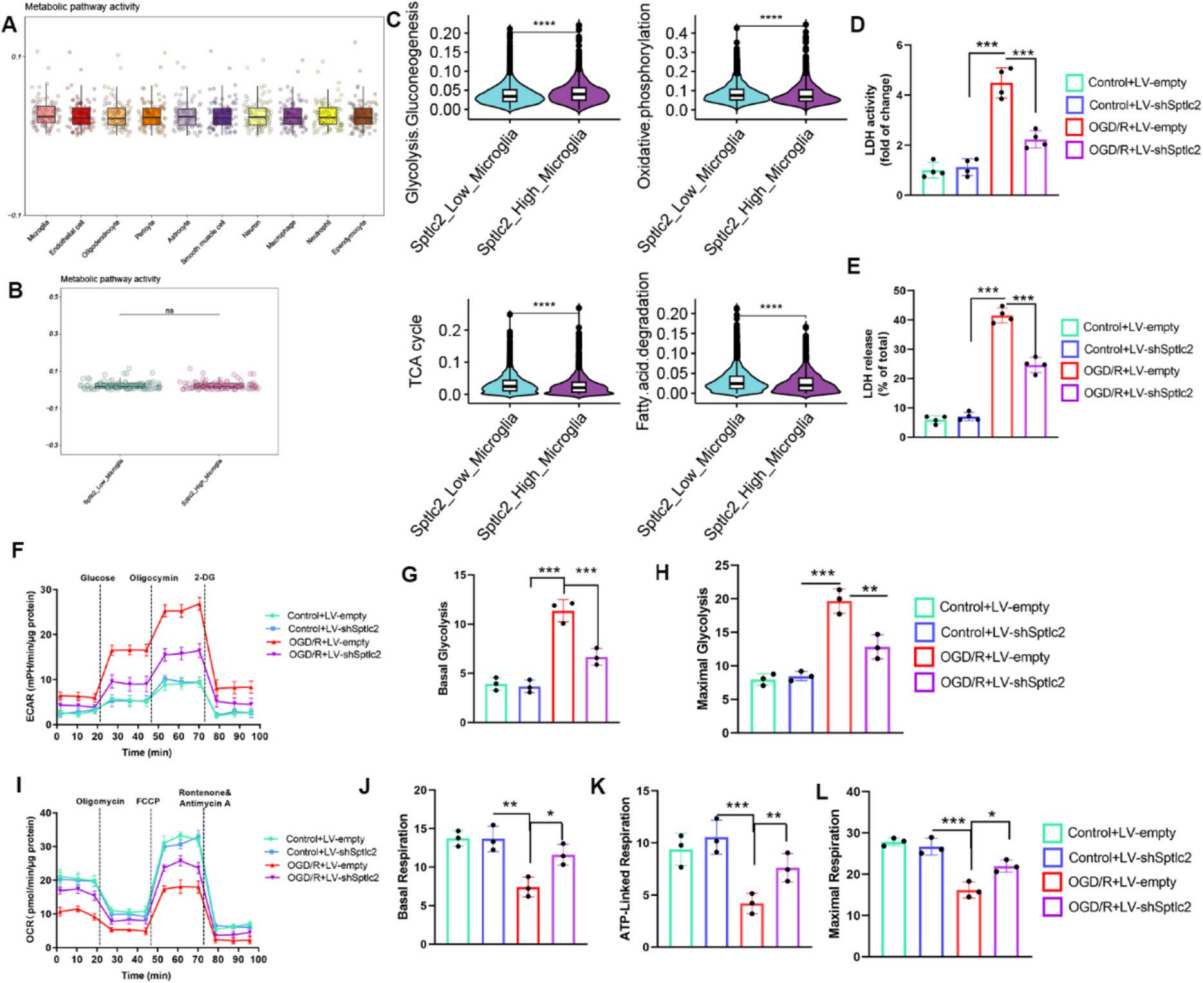
SPTLC2 knockdown boosts OXPHOS while reducing glycolysis in microglia after OGD/R. (**A**) A boxplot illustrating the activity of metabolic pathways across all cell populations. (**B**) A boxplot showing metabolic pathway activity differences between microglia with high and low *Sptlc2* expression following tMCAO. (**C**) Violin plots demonstrating the score differences for OXPHOS, glycolysis, TCA cycle, and fatty acid breakdown in *Sptlc2*_Low vs. *Sptlc2*_High microglia. Student's t-test shows ^****^p < 0.0001 vs. the *Sptlc2*_Low_Microglia group. (**D**, **E**) Quantitative results for LDH activity (**D**) and intracellular lactate release (E) in different microglial groups; n = 4 per group. Values are shown as mean ± SD. One-way ANOVA indicates ^***^p < 0.001 vs. the Control + LV-sh*Sptlc2* or Control + LV-empty groups. (**F**) Typical extracellular acidification rate (ECAR) curves for different microglial groups from Seahorse analysis. (**G**, **H**) Quantitative measurements of basal (**G**) and maximal (**H**) glycolysis; n = 3 per group. Values are shown as mean ± SD. One-way ANOVA indicates ^**^p < 0.01, ^***^p < 0.001 vs. the Control + LV-sh*Sptlc2* or Control + LV-empty groups. (**I**) Typical oxygen consumption rate (OCR) curves for different microglial groups from Seahorse analysis. (**J**-**L**) Quantitative data for basal respiration (**J**), ATP synthesis (**K**), and maximal respiration (**L**); *n* = 3 per group. Values are expressed as mean ± SD. One-way ANOVA shows ^*^p < 0.05, ^**^p < 0.01, ^***^p < 0.001 vs. the Control + LV-sh*Sptlc2* or Control + LV-empty groups

It was found that SPTLC2 is a subunit of serine palmitoyltransferase (SPT), which is the rate-limiting enzyme in sphingolipid biosynthesis (Lee et al. 2017). Previous studies have indicated that metabolic disorders maybe crucial pathological factors leading to the progression of stroke (Loppi et al. 2023; Li et al. 2024). However, the complex metabolic heterogeneity and phenotypic biology associated with stroke remain incompletely understood. To investigate the global metabolism of each cell type in I/R, we employed the scMetabolism algorithm to calculate the scores of all 85 active metabolic pathways. Among the major cell types examined, microglia consistently exhibited higher metabolic activity scores (Fig. 9A). While no significant difference was observed in the total metabolic pathway activity between *Sptlc2*_Low and *Sptlc2*_High microglia (Fig. 9B). Consequently, we further explored the internal metabolic heterogeneity within these two groups of microglial subpopulations. Interestingly, we identified a total of 15 differentially expressed metabolic pathways between *Sptlc2* high- and low-expressing microglia.  An increased glycolytic activity, along with decreased oxidative phosphorylation, TCA cycle activity, and fatty acid degradation, was observed in microglia with high *Sptlc2* expression (Fig. 9C).

To validate the results of our single-cell analysis, we evaluated the impact of SPTLC2 on the reprogramming of microglial glucose metabolism following OGD/R injury. Initially, we assessed the glycolytic flux in microglia after SPTLC2 knockdown, revealing that such inhibition significantly reduced lactate dehydrogenase (LDH) activity and intracellular lactate levels post-OGD/R (Fig. 9D-E). Seahorse bioenergetics analysis further illustrated that SPTLC2 knockdown markedly diminished the OGD/R-induced increases in both baseline and stress extracellular acidification rates (ECAR) (Fig. 9F-H). Subsequently, we investigated the oxidative phosphorylation (OXPHOS) activity in microglia following SPTLC2 knockdown. Seahorse analysis indicated that OGD/R injury led to an enhancement in microglial non-mitochondrial, basal, ATP-related, and maximal respiratory rates. However, the inhibition of SPTLC2 in microglia counteracted these effects (Fig. 9I-K). Collectively, these findings suggest that SPTLC2 knockdown reduces glycolytic activity while promoting OXPHOS metabolism in microglia after OGD/R injury.

### Knockdown of SPTLC2 alleviates OGD/R-induced mitochondrial dysfunction

**Fig. 10:**
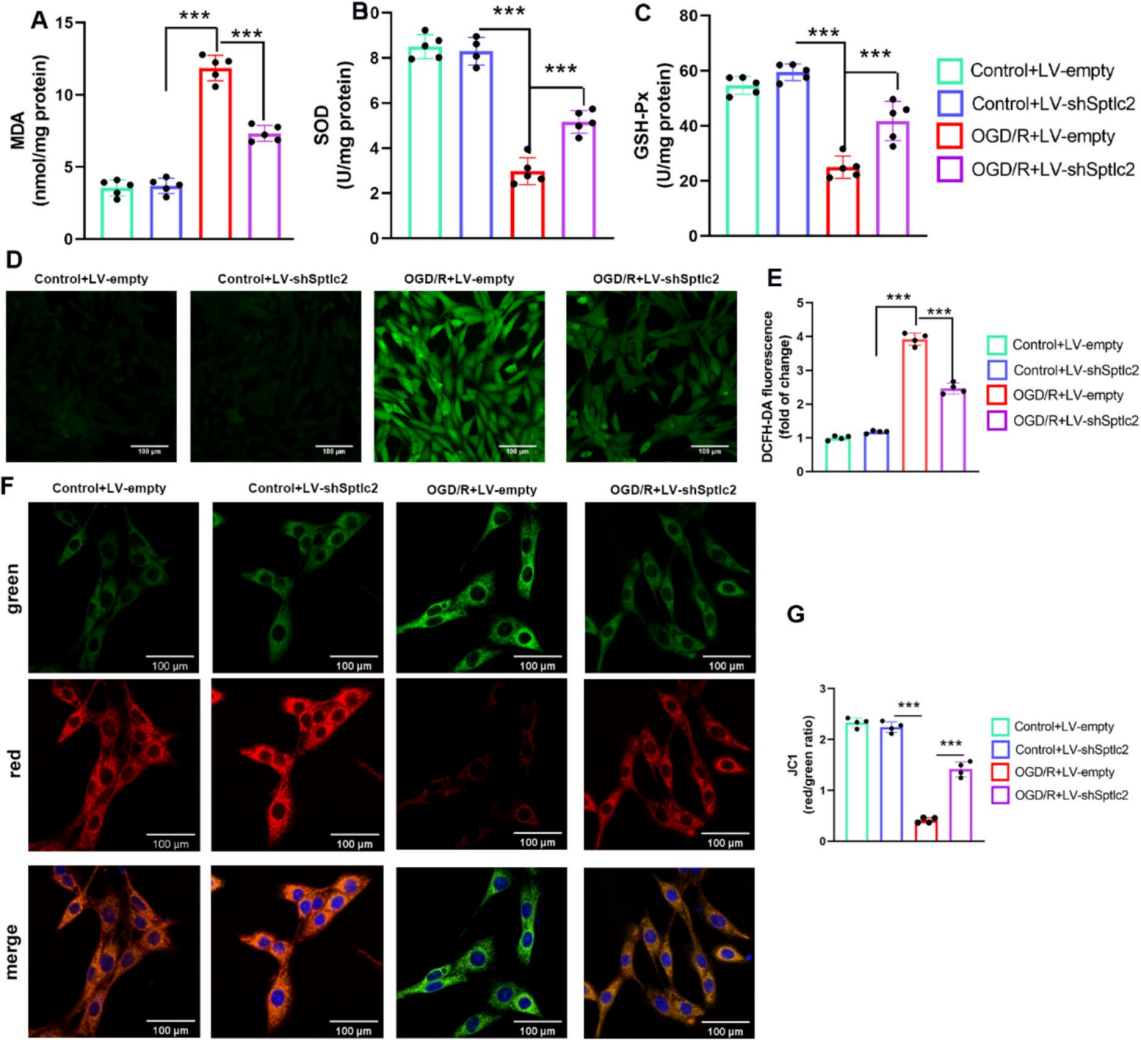
SPTLC2 knockdown mitigates mitochondrial damage in microglia exposed to OGD/R. (**A**-**C**) Quantitative measurements of MDA content (**A**), SOD activity (**B**), and GSH-Px activity (**C**). (D) Microglia from each cohort were stained with DCFH-DA dye, with ROS levels visualized by immunofluorescence (Scale bar = 100 µm). (**E**) Quantitative analysis of ROS fluorescence signal strength. (**F**) Microglia from each group were treated with JC-1 dye, and MMP levels were assessed by immunofluorescence. Red fluorescence indicates the aggregate form of JC-1 in mitochondria with healthy MMP, while green fluorescence shows the monomeric form, indicating MMP dissipation (Scale bar = 100 µm). (**G**) Quantitative analysis of the red-to-green fluorescence intensity ratio; n = 4 per group. All values are shown as mean ± SD. One-way ANOVA indicates ***p < 0.001 vs. the Control + LV-sh*Sptlc2* or Control + LV-empty

Mitochondrial dysfunction is linked to cell death following ischemic insult, which exacerbates brain damage. To elucidate the impact of SPTLC2 on mitochondrial function after cerebral I/R injury, we initially evaluated the oxidative stress levels. The OGD/R injury resulted in increased MDA and overall intracellular ROS levels in microglia but reduced SOD and GSH-Px activities. Inhibiting SPTLC2 partially reversed the above effects (Fig. 10A-E). The mitochondrial membrane potential (MMP) serves as an indicator of mitochondrial functional status, and the preservation of membrane potential is a crucial characteristic of healthy mitochondria. Accumulation of ROS can induce depolarization of MMPs, contributing to mitochondrial damage. Consequently, we evaluated MMP through immunofluorescence using JC-1 dye. As anticipated, MMP depolarization occurred in OGD/R-induced microglia. However, it was preserved following OGD/R injury with SPTLC2 knockdown (Fig. 10F-G). These results suggest SPTLC2 exacerbates microglial injury after OGD/R by elevating ROS levels and mitigating MMP depolarization.

### SPTLC2 induces the alternations of cell–cell communications following I/R injury

**Fig. 11:**
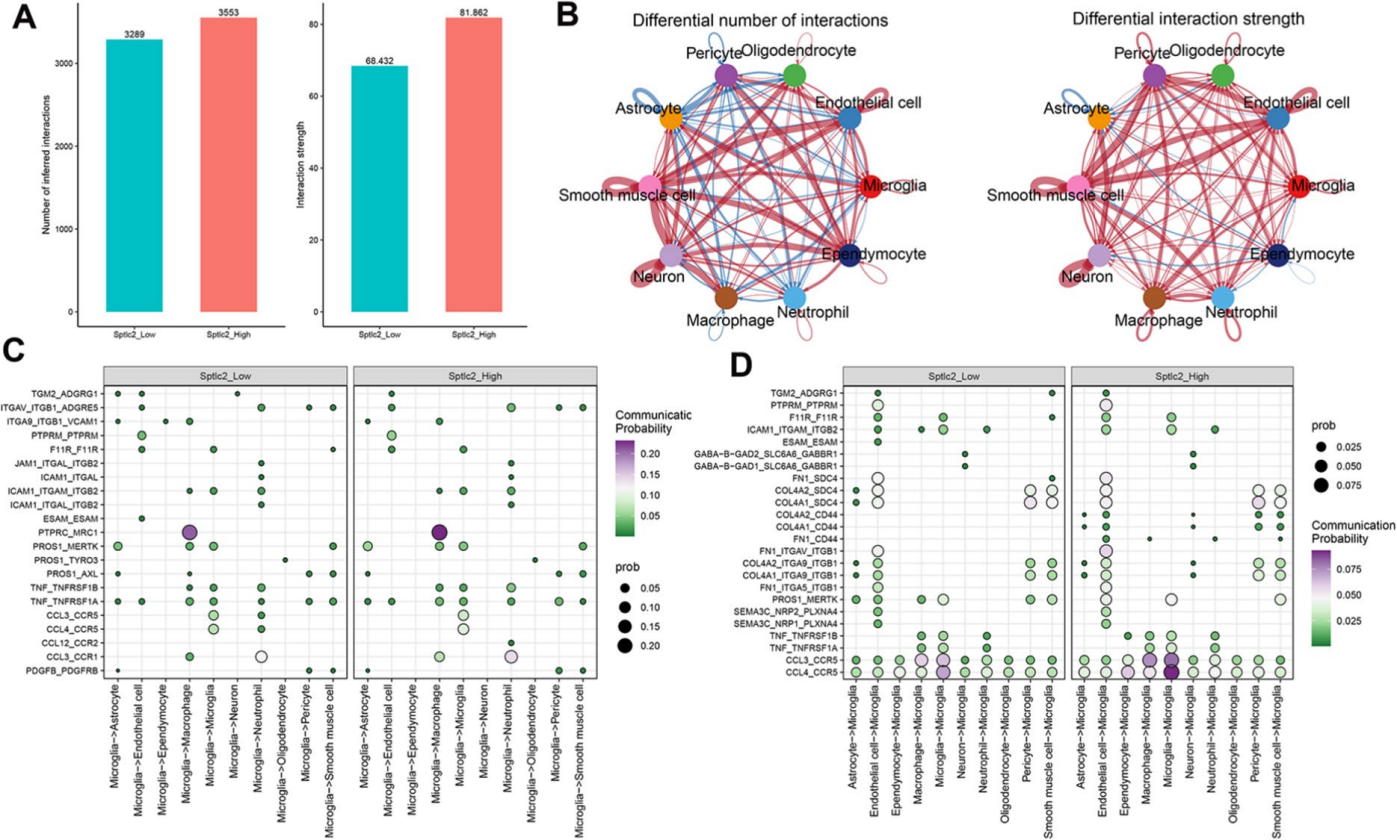
SPTLC2-dependent alterations in cell-to-cell communication during cerebral I/R. (**A**) A bar chart illustrating the quantity and intensity of interactions between major immune cell populations. (**B**) A circle plot contrasting the quantity and strength of intercellular interactions across multiple cell types between the *Sptlc2*_Low and *Sptlc2*_High cohorts. A blue line signifies reduced communication in the *Sptlc2*_High group, while a red line denotes increased communication. The line's thickness represents the number of ligand-receptor pairs. (**C**, **D**) Dot plots showing the outgoing (**C**) and incoming (**D**) receptor-ligand interaction pairs for microglia, comparing *Sptlc2*_Low and *Sptlc2*_High groups

To investigate the impact of SPTLC2 on the molecular interactions among various cell types following injury, we employed CellChat to construct an intercellular communication network based on established ligand-receptor pairs and their cofactors (Plastira et al. 2020). Interestingly, the *Sptlc2* high-expression group exhibited a greater number and intensity of cell–cell interactions compared to the low-expression group, particularly among smooth muscle cells, endothelial cells, neurons, ependymal cells, and other cell types (Fig. 11A-B), indicating enhanced interactions among these cells. Subsequently, we visualized cellular communication across different subpopulations. By analyzing the overall information flow between groups, we identified over 100 signaling pathways, the majority of which were enriched in the *Sptlc2* high-expression group (Figure S8A). Importantly, some pathways associated with stroke prognosis were enriched in the *Sptlc2* high-expression group. For instance, previous studies have demonstrated that acute increases in TNF and cGAS-STING can trigger an inflammatory cascade, resulting in secondary damage following cerebral ischemia (Ding et al. 2005; Chauhan and Kaundal 2023a, 2023b). We further examined the differences in the GAS and SPP1 signaling pathway networks among each cell type between the *Sptlc2*_Low and *Sptlc2*_High groups at the single-cell level, revealing a significant enhancement of GAS-dependent signals transmitted from smooth muscle cells, macrophages, and ependymal cells to other cell types in the *Sptlc2* high-expression group (Figure S8B-C).

To gain insight into the underlying mechanism by which SPTLC2 affects cellular interactions following I/R injury, we conducted a detailed analysis of changes in receptor-ligand pair levels between two groups. Stronger interaction scores indicate a higher predicted interaction between the two cell types via specific ligand-receptor pairs. Our focus was on the interactions between microglia and other cell subsets. Analysis of the *Sptlc2*_High group revealed significant chemokine signaling pathways originating from microglia, including CCL3-CCR5, CCL4-CCR5, CCL12-CCR2, and CCL3-CCR2 from microglia. Additionally, high expression of *Sptlc2* markedly enhanced PROS and TNF signals produced by microglia, while proliferation-related signaling pathways in the *Sptlc2*_High group, such as PTN-SDC4, PTN-SDC3, PTN-SDC2, and PTN-PTPRZ1, were significantly reduced. Conversely, the crosstalk of other subtypes with microglia demonstrated increased activity in secretory protein pathways such as PROS-MERTK, SEMA3C-(NRP2 + PLXNA4), SEMA3C-(NRP1 + PLXNA4), CCL3-CCR5, and CCL4-CCR5 within the *Sptlc2*_High group. In contrast, a reduction in the activity of inhibitory neurotransmitter receptors (GABA-B), muscarinic receptor antagonists (LAMA2), and integrin-related signaling (VTN) was observed in the *Sptlc2*_High group **(**Fig. 11C-D**)**. Collectively, these findings indicate that SPTLC2 serves as a crucial role in affecting the prognosis of ischemic stroke through regulating complicate cell interactions.

### Identification of SPTLC2-associated transcription factors in microglia

**Fig. 12:**
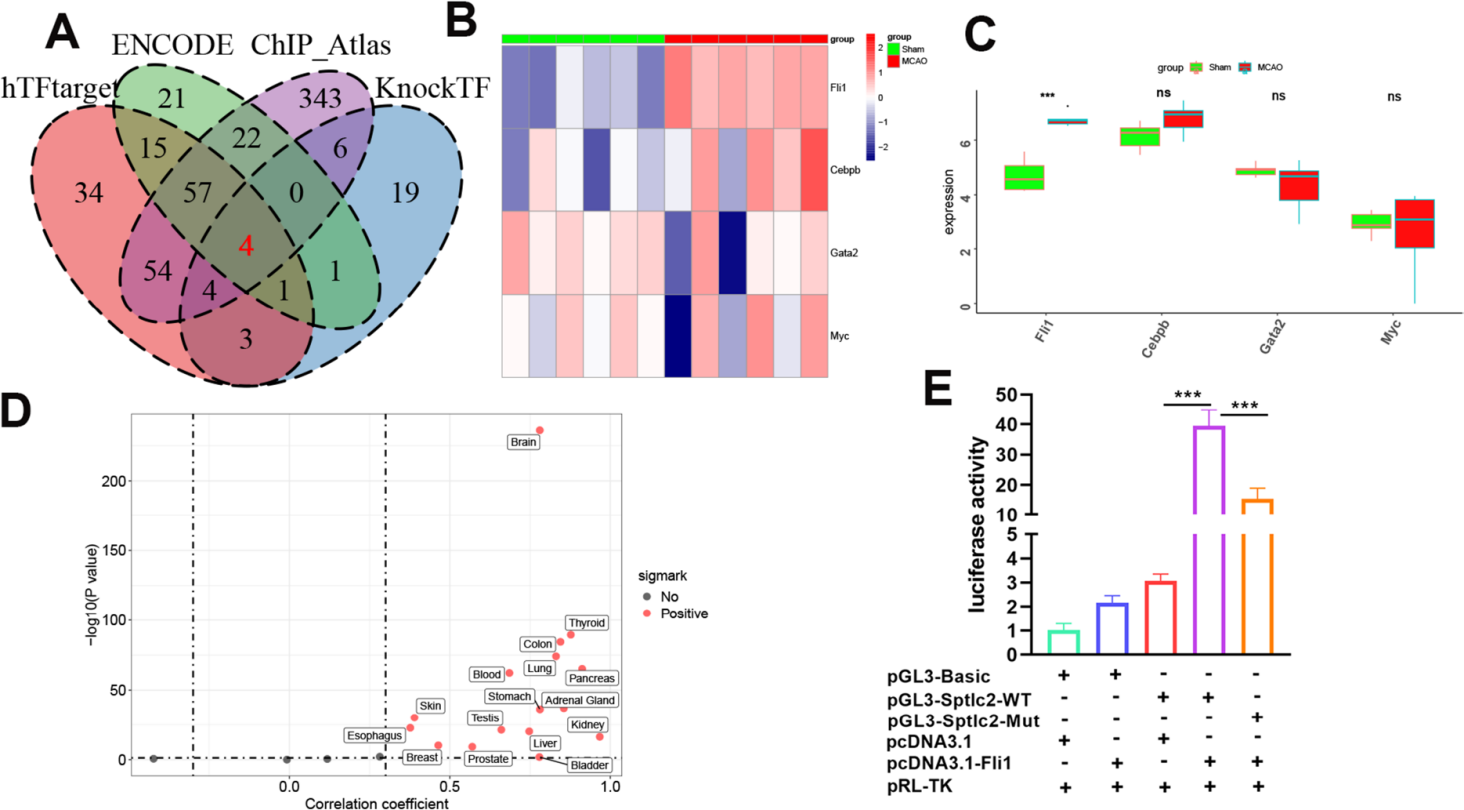
The transcription factor FLI1 regulates SPTLC2. (**A**) Predictions from four bioinformatics tools identifying upstream TFs that target SPTLC2. (**B**, **C**) A heatmap and bar graphs showing expression differences for four predicted TFs (*Fli1*, *Cebpb*, *Gata2*, *Myc*) between Sham and MCAO cohorts in the GSE137482 dataset. (**D**) A correlation plot between SPTLC2 and FLI1 expression from the Genotype-Tissue Expression (GTEx) database. (**E**) Results from luciferase reporter assays confirming that *Fli1* binds to the *Sptlc2* promoter, inducing its expression; n = 3 per group. Values are shown as mean ± SD. One-way ANOVA shows.^***^p < 0.001

Considering the critical role of transcription factors (TF) in regulating gene expression, we therefore utilized four TF prediction websites to elucidate the comprehensive upstream regulatory mechanism of SPTLC2. By combining the result of each dataset, we found four co-regulated TFs (CEBPB, FLI1, GATA2, and MYC) (Fig. 12A). Further analysis demonstrated that only *Fli1* expression was significantly up-regulated in mice after MCAO injury from the GSE137482 dataset (Fig. 12B-C). Therefore, FLI1 was selected for further study. We then utilized the GTEx database to perform correlation analysis on SPTLC2 and FLI1, and the results showed that they were highly positively correlated in various tissues including the brain (Fig. 12D, r= 0.78, P < 0.0001). Luciferase assays were subsequently conducted by cloning mutated –36 to –53 bp fragments of *Sptlc2* promoter (pGL3-*Sptlc2*-Mut), which abrogated the putative *Fli1*-binding sites, and wild-type fragment sequence (pGL3-*Sptlc2*-WT) as control. As a result, the enhanced luciferase promoter activity was observed when transfected pGL3-*Sptlc2*-WT into *Fli1*-overexpressed BV2 cells, while transfection with pGL3-*Sptlc2*-Mut significantly inhibited luciferase promoter activity (Fig. 12E). These results suggested that FLI1 promoted SPTLC2 transcription in microglia.

### Prediction of SPTLC2 target potential drugs based on the molecular docking

**Fig. 13:**
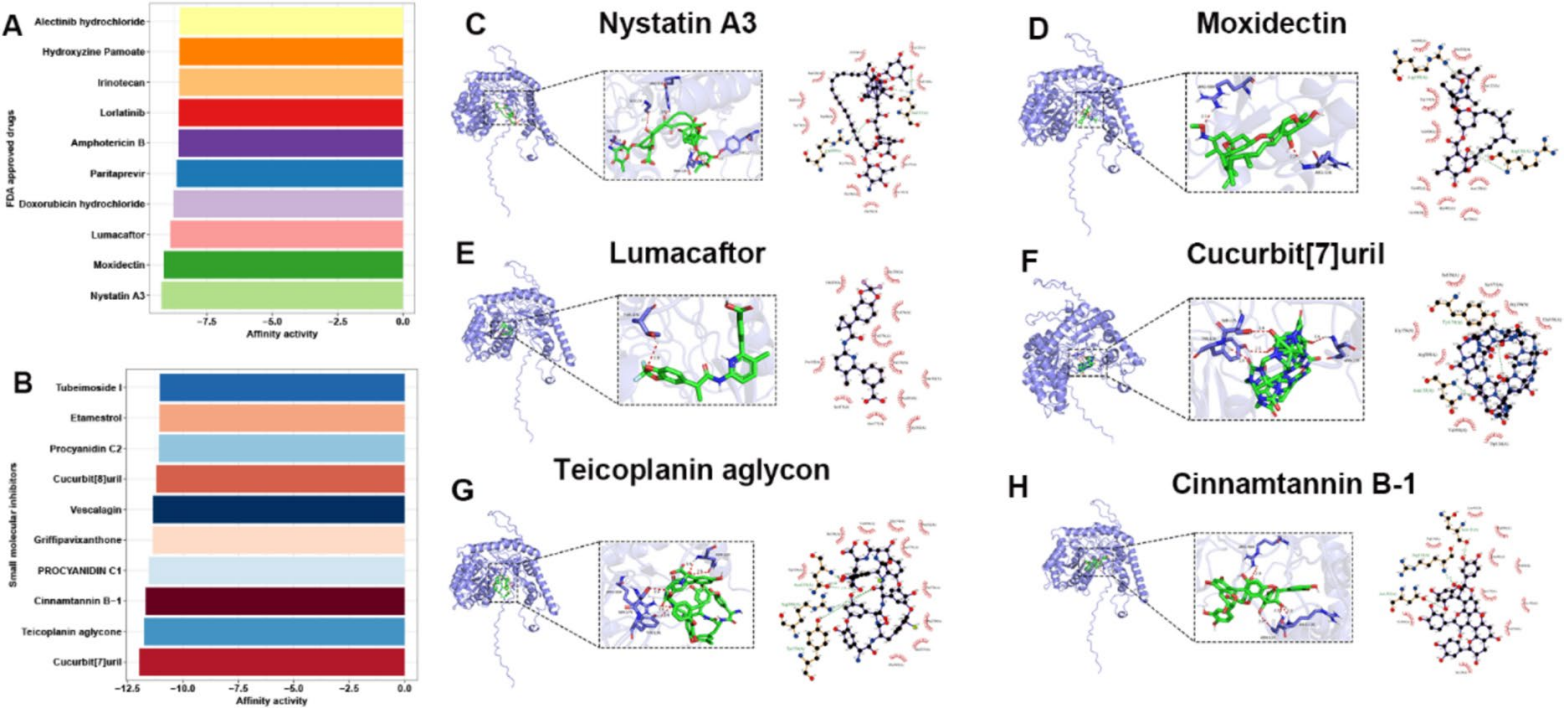
Screening for FDA-approved drugs and small molecules that target SPTLC2. (**A**, **B**) Heatmaps illustrating the docking affinity scores between SPTLC2 and a panel of FDA-approved drugs (**A**) or small molecule compounds (**B**). (C-E) Visualizations of the molecular docking poses for the top three FDA-approved drugs with the strongest affinity scores. (**F**–**H**) Visualizations of the molecular docking poses for the top three small molecule compounds demonstrating the highest affinity scores

To further investigate potential drug candidates targeting SPTLC2, we performed the molecular docking of the SPTLC2 protein structure with over 50,000 small-molecule compounds and 1891 FDA-approved drugs. This analysis aimed to identify compounds with inhibitory potential against SPTLC2. Using the PrankWeb tool, multiple potential molecular docking pockets were identified based on the predicted structure of the SPTLC2 protein. The pocket with the highest predicted score was selected to perform subsequent docking  processes. Furthermore, the Uni-Dock was performed to calculate the affinity scores between SPTLC2 and various compounds. Lower affinity scores represent stronger binding ability between compounds and the protein. We found FDA drugs, including Nystatin A3, Moxidectin, and Lumacaftor exhibited the highest affinity, with scores of −9.236, −9.149, and −8.899. While among small molecular compounds, Cucurbit[7]uril, Teicoplanin aglycone, and Cinnamtannin B-1 represented the top three affinity, with scores of −11.973, −11.739, and −11.688, (Fig. 13A-B). Figure 13C-H exhibited the binding poses and sites.

## Discussion

In the current study, we investigated the gene signatures and potential biological functions associated with IS by integrating bulk and scRNA-seq datasets, emphasizing the role of MRGs in IS prediction and therapy. We employed WGCNA to identify two crucial gene modules (yellow and blue) that were closely associated with IS. These modules encompass 5348 genes and are enriched in processes related to immune response, nerve regeneration, plasma membrane trafficking, cytokine production, and chemokine receptor activity. Machine learning-based integration revealed 18 MRGs significantly associated with IS, of which six MRGs (ELAC2, SPTLC2, MRPL12, NIPSNAP1, IVD, and ABHD11) demonstrated high diagnostic potential, particularly noting that SPTLC2 expression increases during IS development. The scRNA-seq analysis confirmed that SPTLC2 was upregulated across various cell types, especially in microglia, endothelial cells, and macrophages. Furthermore, SPTLC2 has been shown to be regulated by the upstream transcription factor  FLI1, which promotes microglial pro-inflammatory phenotype and mitochondrial dysfunction, increases sensitivity to glycolysis, drives complex cellular communication patterns, and ultimately contributes to cerebral ischemia and reperfusion injury. Finally, molecular docking studies identified potential drugs targeting SPTLC2, including  Nystatin A3, Moxidectin, and Lumacaftor, suggesting their therapeutic potential for IS.

The utility of WGCNA facilitates the identification of co-expression modules, thereby enhancing our understanding of the gene regulatory networks implicated in disease progression. This methodology captures the intricacies of gene interactions that may not be evident in traditional differential expression analyses (Zong et al. 2023; Wang et al. 2022; Sun et al. 2023b). In our study, we identified two key modules (yellow and blue) that exhibit a remarkable correlation with IS, (R exceeding 0.7 and p-value less than 0.05). Furthermore, genes within these two modules demonstrated positive correlations between GS and MM, indicating their potential as functional gene sets for IS. Notably, despite the successful identification of two co-expressed gene modules related to IS, their biological significance remains ambiguous. Previous studies have highlighted several biological processes and pathways related with IS; for instance, some researchers have underscored the role of neuroinflammation, particularly the involvement of immune responses and cytokine production (Jayaraj et al. 2019; Anthony et al. 2022; Candelario-Jalil et al. 2022). Similarly, other studies have discussed the impact of the NF-kappa B signaling pathway in IS progression (Li et al. 2023a; Gao et al. 2023). Consistent with these findings, our study also revealed through enrichment analysis that genes in the identified modules were highly correlated with immune response, cytokine production, and chemokine receptor activity. However, the WGCNA approach offers a more detailed and comprehensive perspective on gene networks, representing a significant advancement over earlier studies that primarily focused on individual genes or pathways.

Machine learning has emerged as a pivotal technology for predicting disease progression and prognosis. Multiple machine learning models integration, namely ensemble learning, possesses several advantages over the use of a single model. First, ensemble methods enhance the robustness and generalization of predictions by reducing overfitting and variance, effectively combining the strengths of multiple models to mitigate the weaknesses inherent in any single model (Bonazzola et al. 2024). Second, these methods are particularly adept at handling complex and high-dimensional data, making them invaluable in fields such as genomics and medical research, where data complexity is prevalent (Wang et al. 2024; Zhang et al. 2024). In this study, we conducted feature selection using a novel machine learning-based ensemble framework. The combination of RF and ridge regression successfully identified 18 key MRGs, with the highest average AUC values across  datasets. ROC curve analysis further revealed 6 MRGs (ELAC2, SPTLC2, MRPL12, NIPSNAP1, IVD, and ABHD11) with robust AUC values in the training (AUC > 0.87) and test group (AUC > 0.72). The high AUC scores and their consistency across different datasets underscore the robustness and reliability of these candidate MRGs as potential diagnostic biomarkers for IS.

Based on bulk RNA sequencing, we found the increased expression of SPTLC2 in IS and its close association with immune cell subsets. SPTLC2 is a key member of the serine palmitoyltransferase (SPT) enzyme complex,  participates in the synthesis of various sphingolipids, including ceramide and its downstream products—sphingosine, sphingosine-1-phosphate, sphingomyelin, and glucosylceramide (Srivastava et al. 2023). Sphingolipids are abundant in nerve cell membranes and myelin sheaths of nerve fibers, associated with multiple cellular functions (Brodowicz et al. 2018; Millner and Atilla-Gokcumen 2021). Several studies have reported that dysregulated sphingolipid metabolism is linked to neurodegenerative diseases and aging (Chakrabarti et al. 2016; Baloni et al. 2022; Trayssac et al. 2018). It has been demonstrated that lipopolysaccharide (LPS) promotes sphingosine biosynthesis by enhancing the expression of SPTLC2, while SPTLC2 deficiency can significantly reduce the production of inflammation-related cytokines (Hering et al. 2024). Furthermore, abnormalities in sphingolipid metabolism were observed in the acute phase of stroke, indicating their disrupted function in mediating brain injury (Zhou et al. 2021; Lucaciu et al. 2022). In contrast to bulk RNA sequencing, scRNA-seq can identify cell-specific gene expression profiles and provide insights into the heterogeneity of cellular responses to ischemic injury, which is critical for understanding the complex cellular interactions in IS. In our study, we characterized the changes in cellular composition of brain tissue following ischemic stroke in mice through scRNA-seq analysis. Our findings revealed that * SPTLC2* was significantly expressed in various cell types, including microglia, highlighting its potential role in the progression of ischemic stroke.

We then integrated molecular, cellular, and behavioral analyses to verify the results derived from the bioinformatics analysis. Our findings indicate that SPTLC2 expression peaks at 3 days post-tMCAO, a critical time point in the evolution of ischemic injury that often marks the height of the post-ischemic inflammatory response and the beginning of a transition towards reparative processes. The dynamic regulation of SPTLC2 suggests it may play a key role in modulating microglial function by sustaining a pro-inflammatory and glycolytic state and hinders the shift towards a more reparative microglial phenotype. Immunofluorescence revealed a sustained increase in SPTLC2 expression in microglia, a key cell type involved in neuroinflammation, suggesting its crucial role in mediating microglial activation and the subsequent inflammatory responses that exacerbate brain damage (Kim et al. 2022; Zhang et al. 2023b; Liu et al. 2020). Furthermore, the observed benefits of SPTLC2 knockdown in reducing neurological deficits, infarct volume, and neuroinflammation support this hypothesis.

Metabolic reprogramming has emerged as a hallmark in the progression of various central nervous system diseases (Devanney et al. 2020; Liu et al. 2024; Gong et al. 2024). Previous studies indicate that the metabolic transformation of microglia is influenced by cellular heterogeneity (Pan et al. 2022; Ma et al. 2023b; Gu et al. 2021). For instance, the pro-inflammatory phenotype is associated with increased glycolysis, while the anti-inflammatory phenotype is characterized by enhanced mitochondrial OXPHOS (Chausse et al. 2021). Currently, there is limited evidence suggesting that microglia in the brains of stroke mice preferentially utilize glycolytic metabolism, although the underlying mechanisms remain unclear (Li et al. 2023b). Therefore, further researches are urgently needed to ascertain the mechanisms and implications of microglial glucose metabolism reprogramming in the context of stroke. In this regard, SPTLC2 has been implicated in regulating sphingolipid metabolism, yet its role in microglia has not been thoroughly investigated. Our study suggests that high SPTLC2 expression is associated with a microglial phenotype characterized by the upregulation of pro-inflammatory genes (Il1b, Tnf) and M1-like markers (CD16), alongside a reduction in M2-like markers (CD206). While our study does not formally demonstrate a state transition, these findings strongly indicate that SPTLC2 promotes a shift toward a pro-inflammatory microglial state, thereby exacerbating neuroinflammation. Future studies employing single-cell trajectory inference analysis could more definitively map how SPTLC2 influences the dynamic progression of microglial activation states after stroke. Notably, by employing an integrated approach that combines single-cell RNA sequencing with experimental validation, we unexpectedly found the critical effects of SPTLC2 on modulating the metabolic state of microglia following brain ischemia/reperfusion injury, characterized by increased glycolytic activity and decreased oxidative phosphorylation, as well as diminished TCA cycle activity and fatty acid degradation. These findings indicate that * SPTLC2* could represent a critical therapeutic target for stroke treatment.

Further, we found FLI1 acts as a transcription factor regulating SPTLC2, which may serve as a critical target for the deterioration of neurological function following brain I/R injury. FLI1 is a member of the ETS family and participate in mediating the progression of various diseases (Mikhailova et al. 2023; He et al. 2021; Chen et al. 2022). The reduced FLI1 expression is observed in brain from human AD cases and 5xFAD mice, as evidenced by the reduced inflammatory response, improved blood–brain barrier (BBB) dysfunction, and decresed amyloid-beta (Aβ) accumulation, which are all critical factors in the progression of AD (Li et al. 2022a). In addition, FLI1 is demonstrated to be involved in the etiology of autoimmune diseases by regulating immune cells and promoting the expression of cytokines and chemokines (Asano et al. 2010; Xu et al. 2016). In this study, we present evidence that SPTLC2 is regulated by FLI1, suggesting their involvement in the modulation of microglial inflammation following cerebral I/R damage.

Molecular docking is a computational technique extensively employed in drug discovery and molecular modeling to predict the preferred orientation of a ligand when binding to a target protein or macromolecule. This approach is essential for elucidating interactions between small molecules and biological macromolecules, thereby aiding in the identification of potential drug candidates (Pinzi and Rastelli 2019; Li et al. 2022b). In this study, we screened and identified three FDA-approved drug candidates that exhibited the highest predicted binding affinity to SPTLC2: Nystatin A3, Moxidectin, and Lumacaftor, along with small molecule compounds such as Cucurbit[7]uril, Teicoplanin aglycone, and Cinnamtannin B-1. Based on the affinity determined through molecular docking, we found that these drugs stably bind to SPTLC2 via the formation of hydrogen bonds, thereby demonstrating the druggability of SPTLC2. It was found that Nystatin may participate in modulating the expression of genes involved in cell survival and proliferation inhibition, including YWHAZ, which has critical anti-apoptotic functions (Asiamah et al. n.d.). Although the primary therapeutic applications of these compounds have been well established, their specific roles in the nervous system have rarely been reported.

Several limitations in our study should be noted. First, though these datasets provide valuable insights, the sample size and cohort diversity may not be sufficient to generalize the findings to a broader population. Future studies should include prospective and larger multi-center cohorts to enhance the robustness of the results. Second, the precise biological mechanism through which SPTLC2 modulates microglial polarization, metabolic reprogramming, and cell–cell communication remains to be fully confirmed and elucidated via additional experiments. Third, the regulatory network of SPTLC2 is likely more complex and may involve multiple transcription factors and epigenetic modifications. Comprehensive analyses, such as chromatin immunoprecipitation sequencing (ChIP-seq) and ATAC-seq, could provide deeper insights into the transcriptional regulation of SPTLC2. Fourth, our study relies on scRNA-seq, which, despite its power in dissecting cellular heterogeneity, requires tissue dissociation and thus loses the spatial context of cellular interactions. The spatial organization of immune and stromal cells within the neurovascular unit is critical to stroke pathophysiology. The advent of spatial transcriptomics technologies offers an exciting opportunity for future studies to map the expression of SPTLC2 and its associated cellular programs in situ, which would provide invaluable insights into how SPTLC2-mediated changes in microglia influence neighboring cells within the ischemic penumbra. Finally, although the molecular docking study offers valuable insights into potential therapeutic targets for cerebral I/R injury, further validation is essential to determine the efficacy of these compounds.

## Conclusion

The study identified two critical gene modules (yellow and blue) strongly correlated with ischemic stroke through WGCNA. Genes within modules were primarily associated with immune response, nerve regeneration, and lipid metabolism. Further, our integrative machine learning-based framework identified key MRGs, which is a combination of RF and Ridge regression models fit on an 18-gene panel. Among these, six MRGs (ELAC2, SPTLC2, MRPL12, NIPSNAP1, IVD, and ABHD11) were validated as potential diagnostic biomarkers for IS, with SPTLC2 showing increased expression in various cell types, including microglia. Functional studies revealed that SPTLC2 promotes microglial activation, metabolic reprogramming, and cell–cell communication alterations, exacerbating ischemic brain injury. Suppression of SPTLC2 alleviated neurobehavioral deficits, reduced infarct volume, and improved mitochondrial function, suggesting its potential as a therapeutic target for cerebral I/R injury. Additionally, the transcription factor FLI1 was identified as a critical regulator of SPTLC2 in microglia, and molecular docking predicted several compounds with potential inhibitory effects on SPTLC2, including Nystatin A3, Moxidectin, and Lumacaftor, as well as Cucurbit[7]uril, Teicoplanin aglycone, and Cinnamtannin B-1. Collectively, we provide a foundation for developing novel therapeutic strategies targeting SPTLC2 to mitigate the detrimental effects of cerebral I/R injury.

## Supplementary Information

Below is the link to the electronic supplementary material.

Supplementary file1 (DOCX 1943 KB)

Supplementary file2 (XLSX 9 KB)

## Data Availability

All of the open-access datasets are available through the GEO database ([https://www.ncbi.nlm.nih.gov/geo/] (https:/www.ncbi.nlm.nih.gov/geo)).
