# Supplementary material for: Characterization of SPTLC2 as a key driver promoting microglial activation and energy metabolism reprogramming after ischemic stroke through bulk and single-cell analyses combined with experimental validation
Source: Cell Biol Toxicol. 2025 Oct 7;41(1):137. doi: 10.1007/s10565-025-10085-9 (PMC12504400; doi:10.1007/s10565-025-10085-9)
Supplement: Supplementary file 1 — Supplementary file1 (DOCX 1943 KB) [file 10565_2025_10085_MOESM1_ESM.docx]

**Characterization of SPTLC2 as a key driver promoting microglial activation and energy metabolism reprogramming after ischemic stroke through bulk and single-cell analyses combined with experimental validation**

**Methods**

**Weighted correlation network analysis (WGCNA)**

To build a co-expression network for pinpointing gene modules linked to clinical statuses (Control and IS), we employed the “WGCNA” software package^[1]^. The process began by selecting the top 50% of genes from the GSE58294 dataset that showed the greatest expression variance. We removed outlier samples before the analysis to fortify the dependability of the scale-free network. The most suitable soft threshold power was chosen by adhering to the scale-free topology standard. A weighted adjacency matrix was then generated and converted into a topological overlap matrix (TOM). From this, a dissimilarity matrix (1-TOM) was calculated. Gene modules were then delineated using hierarchical clustering, setting a minimum size of 150 genes per module. For easier identification, every resulting module was given a distinct color. A Pearson correlation test was performed to assess the correlation coefficient between the discovered modules and clinical states, thereby confirming their biological importance.

**Functional enrichment analysis**

To decipher the functional purposes and signaling routes connected to key genes in the blue and yellow modules, we carried out Gene Ontology (GO) and Kyoto Encyclopedia of Genes and Genomes (KEGG) pathway enrichment studies with the “clusterProfiler” R package ^[2]^. Enrichment terms were deemed significant if their q-value was below 0.05.

To probe functional differences among cell types, we computed the mean gene expression level for each cell with the “AverageExpression” function, after which cells with zero average expression were discarded. We obtained the "hallmark gene sets" from the Molecular Signatures Database (MSigDB) and ran a Gene Set Variation Analysis (GSVA) to appraise functional heterogeneity among different cellular groups^[3]^.

**Cerebral ischemia/reperfusion (I/R) injury in mice.**

We procured male C57BL/6J mice, aged 8-10 weeks and weighing 22-25 g, from the Animal Model Center at Fujian Medical University. These animals were cohabitated in groups of four to five in a specific-pathogen-free (SPF) environment. This facility adhered to a 12-hour light-dark schedule (lights on at 07:00), with regulated temperature (22±2°C) and moisture levels (55±5%). Access to standard rodent food and water was unrestricted. The transient middle cerebral artery occlusion (tMCAO) procedure was executed as described in prior reports^[4]^. Anesthesia was induced with an intraperitoneal injection of 1% sodium pentobarbital (45 mg/kg), and core body temperature was rigorously kept at 37.0±0.5°C during the entire operation. The common carotid artery (CCA) along with the internal carotid artery (ICA) were cautiously exposed. A 6-0 monofilament nylon suture with a silicone coating (Doccol, MA, USA) was guided through the ICA until it reached the origin of the middle cerebral artery (MCA), inducing a 90-minute occlusion. Reperfusion was started by retracting the filament. For the sham-operated control animals, identical surgical steps were taken, but the suture was not advanced into the artery. All mice were given subcutaneous buprenorphine (0.05 mg/kg) for pain relief right after the operation and again every 12 hours for the first two days post-tMCAO.

To measure the volume of cerebral infarcts, animals were euthanized with an overdose of 1% sodium pentobarbital three days after MCAO. Their brains were quickly harvested, cut into 2-mm coronal slices, and bathed in 2,3,5-triphenyltetrazolium chloride (TTC, Sigma, USA) at 37°C for ten minutes. The sections were then fixed for 30 minutes in 4% paraformaldehyde. The infarct volume was calculated with ImageJ software using the formula:

$$\frac{(contralateral hemisphere volume-non-lesioned ipsilateral hemisphere volume)}{the contralateral hemisphere volume}*100\%$$

**Transfection with Lentivirus.**

Lentiviral vectors expressing *Sptlc2*-targeting shRNA (LV_*shSptlc2*) and a non-targeting control shRNA (LV_shNC) were obtained from Hanheng Biotech (Shanghai, China). In brief, primary microglial cells were cultured in 6- or 12-well plates and then infected with the respective lentivirus at a multiplicity of infection (MOI) of 10. After an 8-hour incubation period, the viral medium was replaced with fresh culture medium. *Sptlc2* knockdown efficiency was evaluated three days post-infection using Western blotting and RT-PCR analyses.

**Quantitative real-time polymerase chain reaction (qRT-PCR)**

We extracted total RNA from both microglia and tissues from the peri-infarct cortex with Trizol reagent (TaKaRa, Tokyo, Japan), following the provided protocol. We synthesized complementary DNA (cDNA) with the RevertAid First Strand cDNA Synthesis Kit (Thermo Fisher Scientific, MA, USA) . qRT-PCR was run on an ABI 7500 Real-Time PCR system (Applied Biosystems, Foster City, USA) using a SYBR Green kit (TaKaRa, Tokyo, Japan). The sequences for the primers used are listed in Supplementary Table 1. We normalized the expression levels of target genes to mouse β-actin expression using 2^−ΔΔCT^ method.

**Western-Blot**

Total protein was isolated from tissues or cultured microglia with RIPA lysis buffer (Beyotime, Jiangsu, China) that was fortified with a protease inhibitor cocktail (Roche, Mannheim, Germany). Following centrifugation, we collected the clear supernatants. Protein amounts were quantified with a BCA Protein Assay Kit (Thermo Fisher Scientific, MA, USA) as per its instructions. Thirty micrograms of protein from each sample were loaded onto 10% SDS-PAGE gels and then transferred to polyvinylidene difluoride (PVDF) membranes (Millipore, MA, USA). These membranes were blocked for two hours at room temperature using a solution of 5% bovine serum albumin (BSA, Sigma-Aldrich, MO, USA). The membranes were then incubated with primary antibodies for SPTLC2 (1:200, Santa Cruz, CA, USA) or β-actin (1:5000, Abcam, Cambridge, UK) overnight at 4°C. After washing them three times, we applied the appropriate HRP-conjugated secondary antibodies (1:5000, Abcam, Cambridge, UK) for one hour at ambient temperature. We detected protein bands using an ECL chemiluminescent substrate kit (Advansta, CA, USA) and captured the images with a chemiluminescence imaging system (Bio-Rad, CA, USA). The relative abundance of the target protein was normalized against β-actin using ImageJ software.

**Assessment of ROS** **generation**

We quantified intracellular reactive oxygen species (ROS) with the DCFH-DA fluorimetric probe (Beyotime, Shanghai, China), adhering to the manufacturer's protocol. In short, microglia were treated with DCFH-DA at 37°C for 20 minutes in darkness. This was followed by three washes with PBS to eliminate any leftover probe. We then immediately visualized cellular fluorescence with a fluorescence microscope equipped with a 40× objective lens. The fluorescence intensity for each well was measured using ImageJ software.

**Mitochondrial Membrane Potential (MMP) Assay**

The MMP was gauged with a JC-1 Assay Kit (Thermo Fisher Scientific, MA, USA), as directed by the supplier. Microglial cells grown in 6-well plates were washed twice with PBS and then stained with 2 µM JC-1 dye for 30 minutes at 37°C while protected from light. After staining, the cells were washed two more times with PBS and observed under a fluorescence microscope with a 40x objective. A change in fluorescence from red to green indicated MMP depolarization..

**SeaHorse assay**

Mitochondrial respiratory function was evaluated with the Seahorse XF Glycolysis Stress Test Kit and the Seahorse XF Cell Mito Stress Test Kit (Seahorse Biosciences, Copenhagen, Denmark). Briefly, we seeded primary microglia (4 × 10⁴ cells/well) into XFe24 cell culture microplates and maintained them according to the supplier's instructions. These plates were then hydrated and left overnight at 37°C in a CO₂-free incubator containing XF Calibration Buffer. For measuring the extracellular acidification rate (ECAR), we prepared assay reagents (10 mM glucose, 2 mM oligomycin, 50 mM 2-deoxy-D-glucose) in glycolysis stress test medium and put them into the sensor cartridge. For the oxygen consumption rate (OCR) assay, the cartridge was loaded with 1 mM oligomycin, 2 mM FCCP, and a 0.5 mM mix of rotenone and antimycin A. Following calibration, the sensor cartridge was fitted onto the cell plate, and we measured OCR and ECAR every 8 minutes for a total of 96 minutes, with automated injection of the compounds. All samples were run in triplicate. OCR and ECAR values were adjusted for protein content and are reported as pmol/min/µg protein and mpH/min/µg protein, respectively.

**Immunofluorescence**

Mice were deeply anesthetized and perfused with PBS, which was followed by fixation using 4% paraformaldehyde. Entire brain tissues were harvested, fixed overnight in 4% paraformaldehyde, and then transferred to a 30% sucrose solution at 4°C until fully dehydrated. Primary microglia grown on coverslips were fixed for 15 minutes with 4% paraformaldehyde and then washed three times with PBS. We prepared 10 µm thick frozen coronal brain sections with a cryostat (Leica, Wetzlar, Germany). To improve antigen detection, sections underwent heat-mediated antigen retrieval in citrate buffer for 30 minutes. This was followed by permeabilization using 0.1% Triton X-100 and a 90-minute blocking step at 37°C with 5% bovine serum albumin (BSA). Both sections and cultured cells were incubated overnight at 4°C with primary antibodies: mouse anti-SPTLC2 (1:50, Santa Clara, CA, USA) and rat anti-Iba1 (1:500, Abcam, MA, USA). After extensive washing, we applied the correct secondary antibodies (Abcam, MA, USA) and incubated the samples for 2 hours at 37°C in darkness. Finally, DAPI (Beyotime, Shanghai, China) was used to stain cell nuclei for 15 minutes. A confocal microscope (Zeiss, Gottingen, Germany) was used to capture immunofluorescence images. For our in vivo quantification, we randomly chose 6-8 microscopic fields from the peri-infarct cortex of each mouse. Our focus was on cortical areas directly bordering the infarct core, which is a site of intense glial activation and secondary damage. An investigator who was unaware of the experimental conditions performed the image analysis with ImageJ software (v1.53, NIH, USA). For co-localization studies, we thresholded individual channels for Iba1, SPTLC2, CD16, or CD206 to separate the signal from the background. For cell counting, data are shown as the count of positive cells per mm^2^ or per 0.1 mm^2^. For polarization studies, data are given as a percentage of all Iba1+ cells. For our in vitro work, we imaged a minimum of six random fields on each coverslip.

**Results**

**
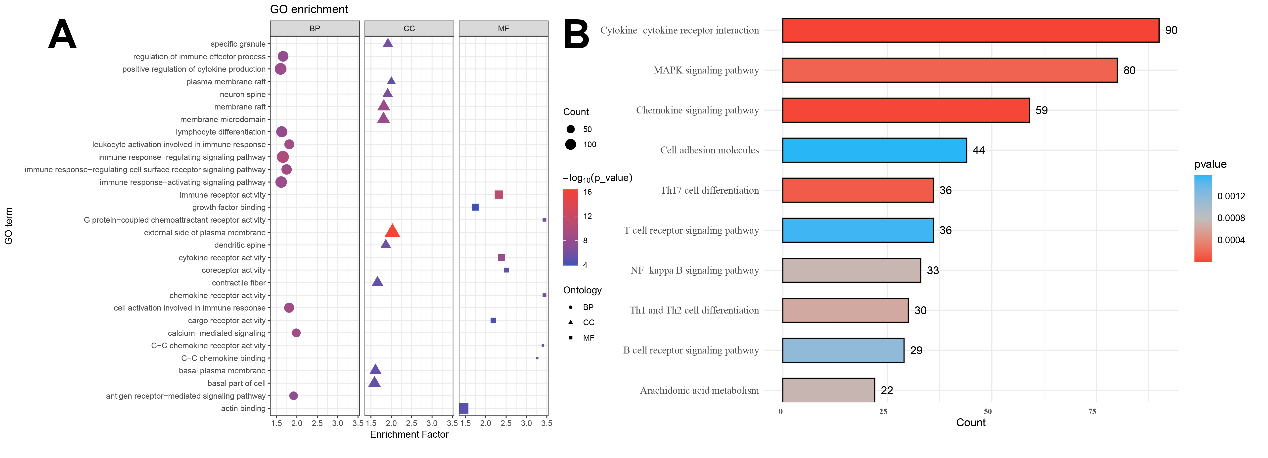
**

**Figure S1 GO and KEGG enrichment analysis of hub genes in the yellow and blue modules.**

**
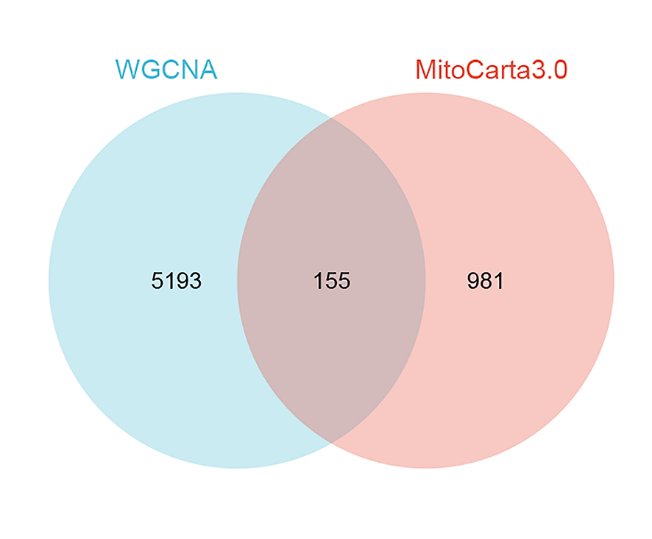
**

**Figure S2 WGCNA and the MitoCarta3.0 dataset co-identifed 155 MRGs**

**
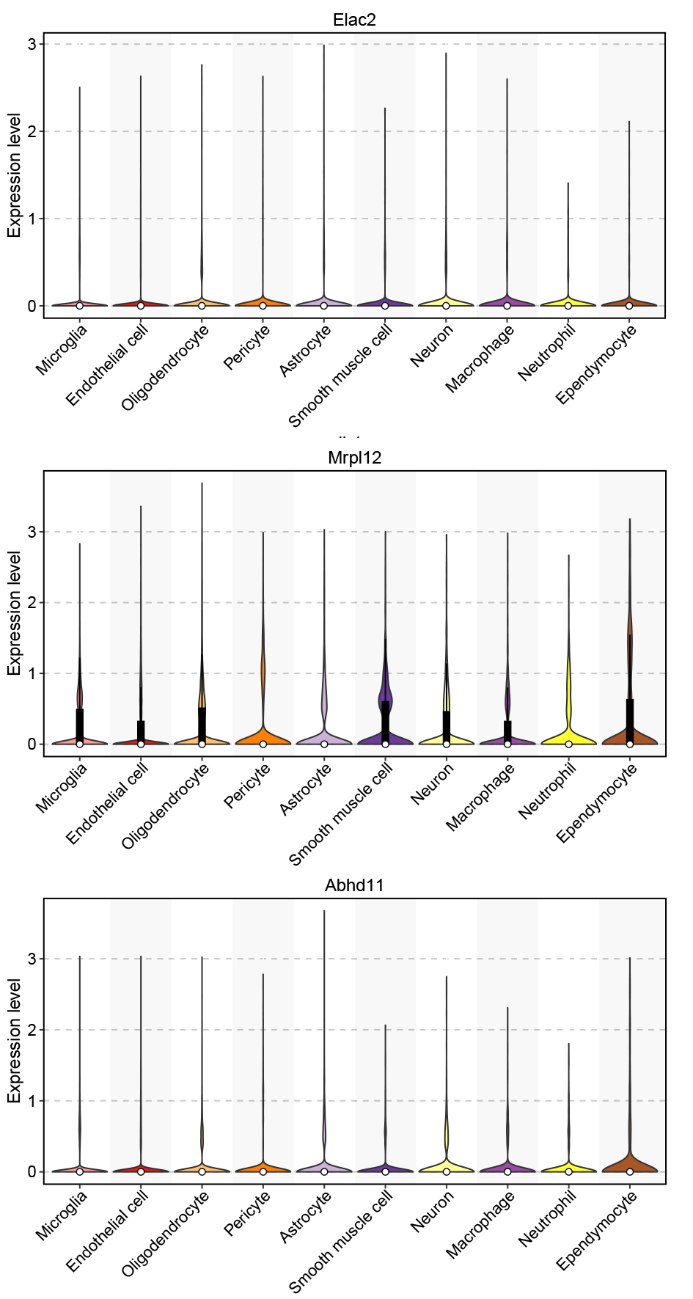
**

**Figure S3 Violin plot deposits the expression differences of Elac2, Mrpl12, and Abhd11 among various cell types at single-cell level.**

**
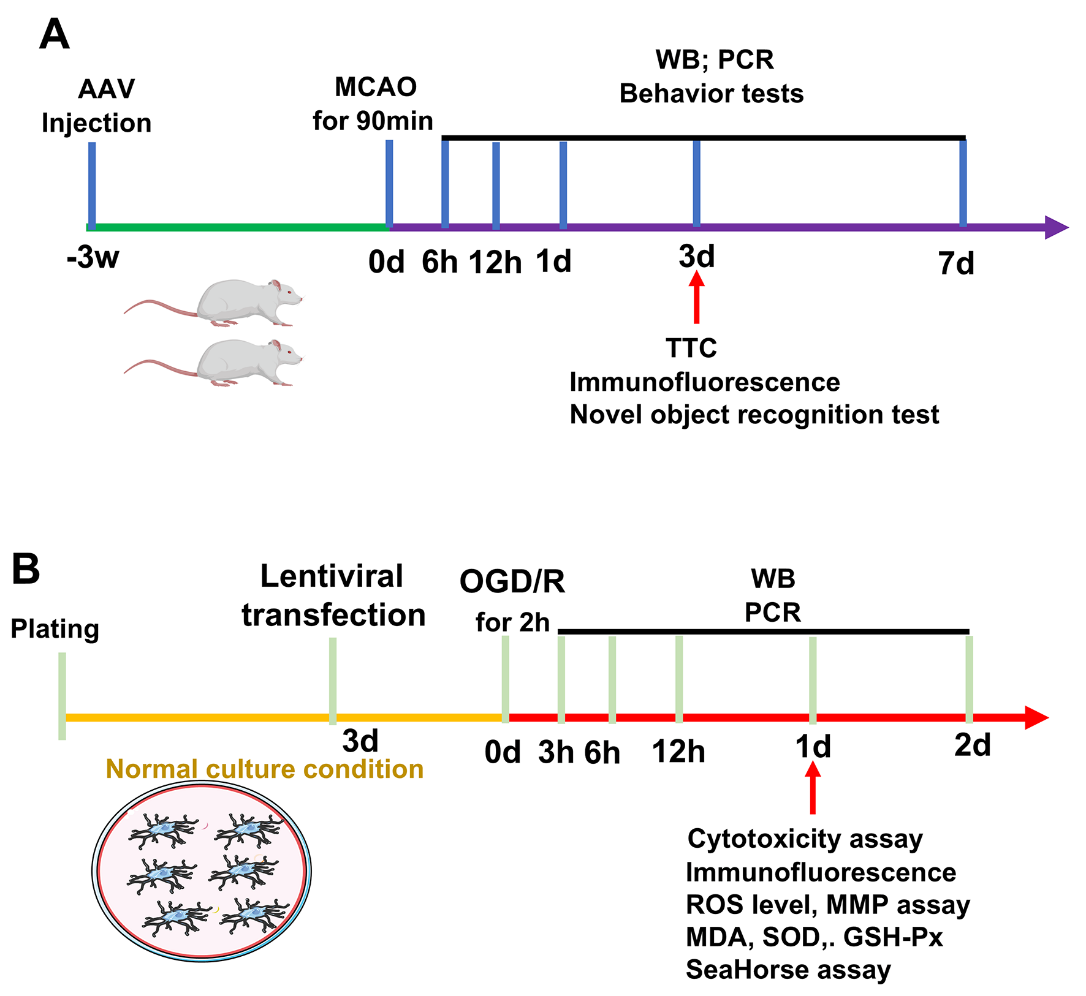
**

**Figure S4 Overall experimental design for in vivo and in vitro validation.**

**
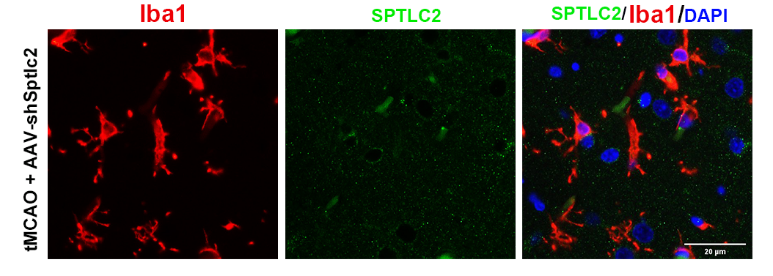
**

**Figure S5 Immunofluorescence validation of SPTLC2 knockdown efficiency.**

**
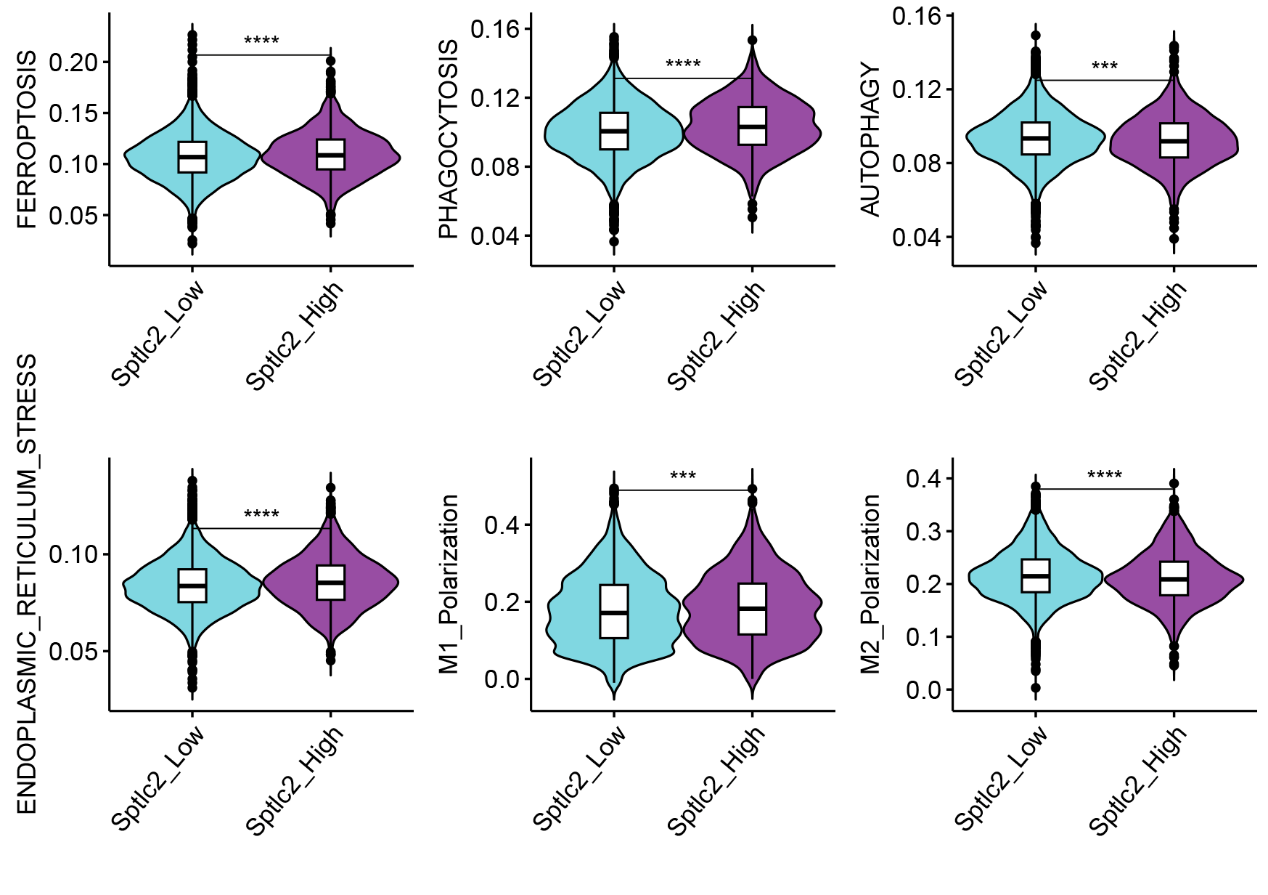
**

**Figure S6 Violin plot illustrates the differences in various phenotypic scores between Sptlc2_Low and Sptlc2_High group.**

**
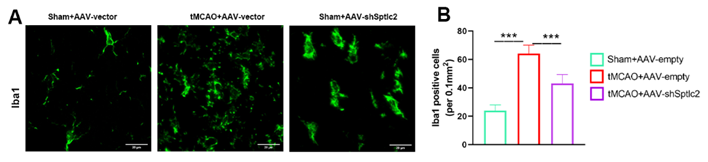
**

**Figure S7. SPTLC2 knockdown attenuates the numbers of microglia after tMCAO.** (A) Representative immunofluorescence images of Iba1 staining (gren) in the peri-infarct cortex used for quantification. (B) Quantification of the total number of Iba1+ cells per 0.1mm² in the peri-infarct cortex of Sham + AAV-empty, tMCAO + AAV-empty, and tMCAO+AAV-shSptlc2 mice. n=4/group. Data are represented as mean ± SD. ^*^*P* < 0.01, ^**^*P* < 0.001.

**
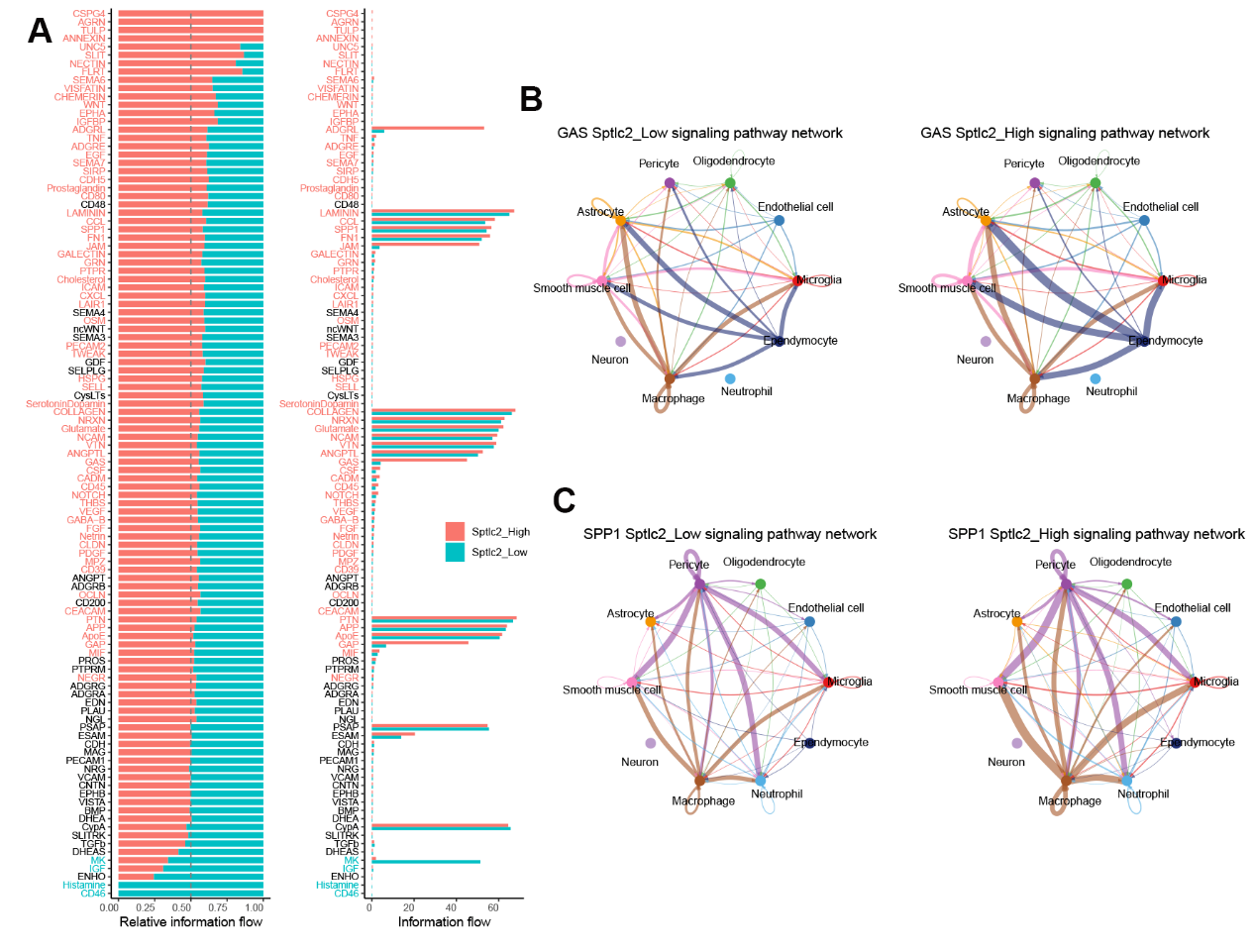
**

**Figure S8 The changes of intercellular communications among various cell types.** (A) Bar plot displaying the overall information flow (left) and differences in the information flow (right) of significant signaling pathways between Sptlc2_Low and Sptlc2_High group. Red indicating top pathways enriched in Sptlc2_Low, while black indicating equally enriched pathways in both Sptlc2_Low and Sptlc2_High group; green indicating enriched pathways in Sptlc2_Low group. (B,C) Inferred differences in GAS (B) and SPP1 (C) signaling network between Sptlc2_Low and Sptlc2_High groups. The thickness of the line representing the communication probability.

Reference

[1] Langfelder P, Horvath S. WGCNA: an R package for weighted correlation network analysis[J]. BMC Bioinformatics, 2008, 9: 559.

[2] Yu G, Wang L G, Han Y, et al. clusterProfiler: an R package for comparing biological themes among gene clusters[J]. OMICS, 2012, 16(5): 284-287.

[3] Hänzelmann S, Castelo R, Guinney J. GSVA: gene set variation analysis for microarray and RNA-seq data[J]. BMC Bioinformatics, 2013, 14: 7.

[4] Xu P, Zhang X, Liu Q, et al. Microglial TREM-1 receptor mediates neuroinflammatory injury via interaction with SYK in experimental ischemic stroke[J]. Cell Death Dis, 2019, 10(8): 555.
